# eIF5A1/RhoGDIα pathway: a novel therapeutic target for treatment of spinal cord injury identified by a proteomics approach

**DOI:** 10.1038/srep16911

**Published:** 2015-11-23

**Authors:** Wei Liu, Fei-Fei Shang, Yang Xu, Visar Belegu, Lei Xia, Wei Zhao, Ran Liu, Wei Wang, Jin Liu, Chen-Yun Li, Ting-Hua Wang

**Affiliations:** 1 Institute of Neurological Disease, The state key laboratory of Biotherapy, Department of Anesthesiology and Translational Neuroscience Center, West China Hospital, Sichuan University, and Collaborative Innovation Center for Biotherapy, Chengdu, 61041, P.R. China; 2 Institute of Neuroscience, Kunming medical University, Kunming 650031, P.R. China; 3 Key Laboratory of Agro-Biodiversity and Pest Management of Education Ministry of China, Yunnan Agricultural University, Kunming, 650000, P.R. China; 4 Department of Neurology, Johns Hopkins School of Medicine, Baltimore, MD, USA

## Abstract

Spinal cord injury (SCI) is frequently accompanied by a degree of spontaneous functional recovery. The underlying mechanisms through which such recovery is generated remain elusive. In this study, we observed a significant spontaneous motor function recovery 14 to 28 days after spinal cord transection (SCT) in rats. Using a comparative proteomics approach, caudal to the injury, we detected difference in 20 proteins. Two of these proteins, are eukaryotic translation initiation factor 5A1 (eIF5A1) that is involved in cell survival and proliferation, and Rho GDP dissociation inhibitor alpha (RhoGDIα), a member of Rho GDI family that is involved in cytoskeletal reorganization. After confirming the changes in expression levels of these two proteins following SCT, we showed that *in vivo* eIF5A1 up-regulation and down-regulation significantly increased and decreased, respectively, motor function recovery. *In vitro*, eIF5A1 overexpression in primary neurons increased cell survival and elongated neurite length while eIF5A1 knockdown reversed these results. We found that RhoGDIα up-regulation and down-regulation rescues the effect of eIF5A1 down-regulation and up-regulation both *in vivo* and *in vitro*. Therefore, we have identified eIF5A1/RhoGDIα pathway as a new therapeutic target for treatment of spinal cord injured patients.

Spinal cord injury (SCI) results in irreversible loss of motor and sensory function, and currently there are no effective treatments for neurological dysfunctions that result from SCI. A major reason for the devastating prognosis of severe CNS injury with regard to functional recovery is neuronal loss and the inability of CNS axons to re-grow after injury. Severe SCI significantly disrupts neuronal circuitry interrupting input from the sensorimotor cortex. Clinically this disruption is manifested in patients as complete palsy below the injury level^1^,^2^,^3^,^4^,^5^,^6^. Spontaneous functional improvements particularly after spinal cord transection (SCT) are very limited and generally occur shortly after SCI^1^,^2^,^7^,^8^,^9^,^10^,^11^,^12^. Wyart *et al.* showed that Kolmer-Agduhr (KA) neurons in zebrafish stimulate neurons of the central pattern generators (CPG), located within the spinal cord, which generate periodic motor commands necessary for rhythmic movements^13^. This data indicates that neurons within the spinal cord have the capacity to control movement. Therefore, understanding the mechanisms that improve neuronal survival and axonal elongation of caudal to SCI can generate novel therapeutic targets for enhancement of motor function following SCI.

Eukaryotic translation initiation factor (eIF5A1), a protein containing the unusual amino acid hypusine, is highly expressed in neonatal brain^14^,^15^,^16^, but its function in the central nervous system (CNS) remains elusive. Our previous work has shown that eIF5A1 plays a crucial role in the SCT rats’ gastrocnemius muscle^17^.

Actin and microtubule dynamics are obligatory downstream effectors of several signaling cascades involved in axonal elongation. Cytoskeletal regulators such as Rho GTPases and proteins that regulate their function can modulate neuronal plasticity by manipulating actin and microtubule dynamics^18^,^19^,^20^,^21^. In a GDP-bound form, GTPases destabilizes actin filaments, and induces neurite formation in PC-12 and N1E-115 cells^22^,^23^. Consistent with this, RhoGDIα steady GDP-bound state of GTPases could increase actin dynamics, thus destabilizing the actin cytoskeleton and allowing microtubules to protrude into the peripheral area of the growth cone that promote cell growth and axonal regeneration^24^,^25^,^26^. Additionally, Rho GDP dissociation inhibitor alpha (RhoGDIα) combined with GTPases regulates fundamental processes of cell biology such as morphogenesis, polarity, movement, and cell division^20^,^21^,^27^.

Here, using a proteomics approach we identify eIF5A1 and RhoGDIα as proteins that are upregulated during rats’ spontaneous motor function recovery. Both of these proteins are in the same signaling pathway that leads to enhanced neuronal survival and axonal regeneration. Reductions of eIF5A1 and RhoGDIα inhibit the neuroplasticity following SCT while overexpression rescues these protective effects. Additionally, we show eIF5A1 upregulates RhoGDIα within this pathway. The present findings therefore indicate a new strategy to treat patients with SCI.

## Results

### A proteomics screen identified 20 proteins whose expression levels related to spontaneous functional recovery in SCT rats

**Figure 1:**
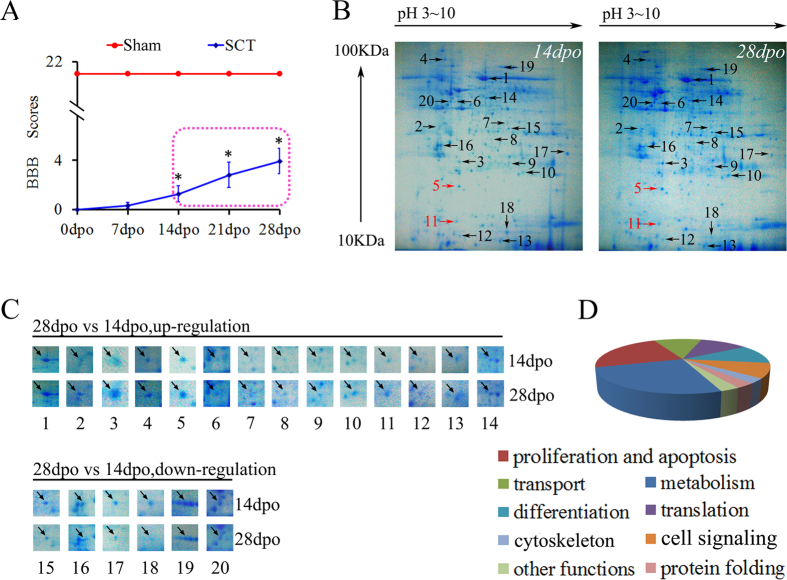
Comparative proteomics analysis of differentially expressed proteins between 14 and 28 days after SCT. (**A**) The hind limb motor function in SCT rats was evaluated using BBB scores 0, 7, 14, 21, and 28 days post-operation (dpo). Uninjured sham controls rats had BBB scores of 21 ± 0.00 at all time points. At 14 dpo to 28 dpo rats showed significant functional recovery (14 dpo vs 7 dpo; 21 dpo vs 14 dpo; 28 dpo vs 21 dpo) (*p < 0.05). (**B**) Representative 2-D maps of samples isolated from the spinal cord caudal of the SCT at 14 dpo and 28 dpo. (**C**) Images of 20 protein spots that contained different protein levels at 14 dpo and 28 dpo. The upper panels contain 14 upregulated proteins while the lower ones contain 6 downregulated proteins. Detailed information of these spots was listed in Table S1. (**D**) Identified proteins categorized into several groups according to their function.

We obtained BBB scores 0, 7, 14, 21 and 28 days post-operation (dpo) in order to evaluate the spontaneous motor function recovery in rats following SCT. On day 14 after SCT, BBB scores became statistically higher compared to BBB scores from day 0 (p < 0.05), and they remained higher 21 d and 28 d after SCT (p < 0.05) (Fig. 1A). Next, we used a proteomics approach to screen proteins that are differentially expressed in the spinal cord caudal to the injury on days 14 and 28 after SCT; experiment was repeated twice. We detected 370 (14 d after SCT) and 358 (28 d after SCT) protein spots by CBB R-250 staining in 2-D gels (Fig. 1B). A total of 20 spots were identified as differentially expressed proteins; of these, 14 proteins were upregulated, while the remaining 6 were downregulated. The fold-change and other detailed information of these spots were listed in Table S1. Spectrum analysis revealed the identity of 18 protein spots but failed to identify two protein spots (scores were lower than 56 as listed in Table S1). Analysis of these proteins showed that 9 proteins are involved in metabolism, 8 in proliferation and apoptosis, 4 in differentiation, 4 in translation, 4 in transport, 3 in signal transduction, 1 in protein folding, 1 in cytoskeleton and 1 has other functions (Fig. 1D).

### Validation of expression of eIF5A1 after SCT

**Figure 2:**
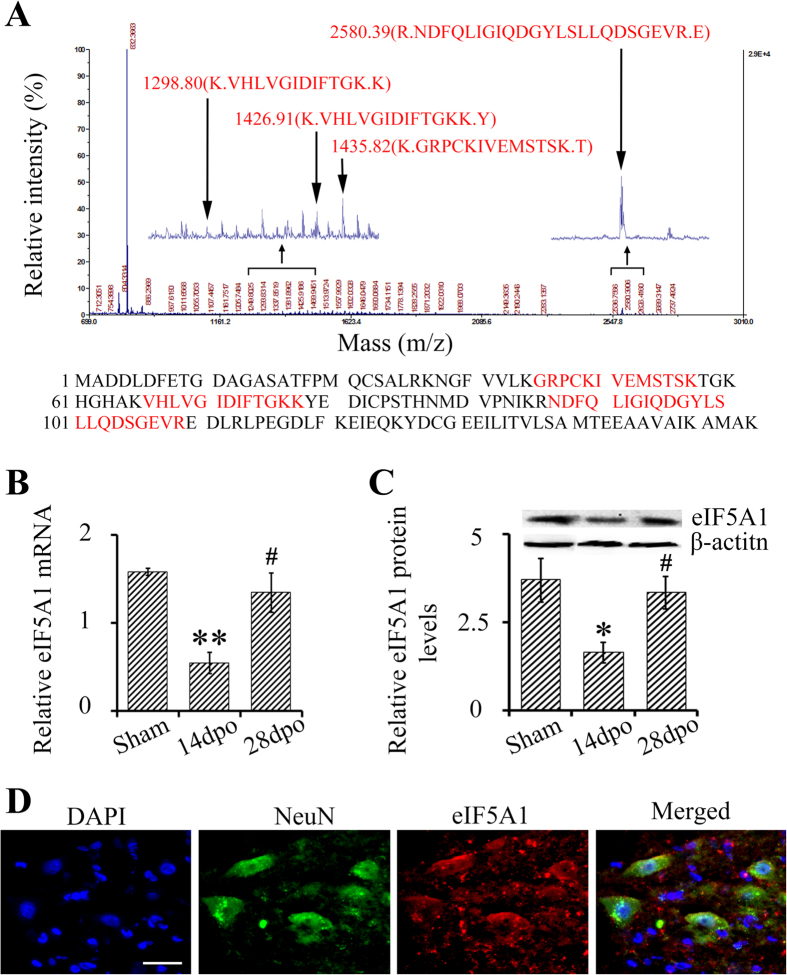
eIF5A1 was upregulated in the spinal cord, caudal to the SCT from 14 dpo to 28 dpo. (**A**) Primary mass spectrometry result of spot 11 (eIF5A1). After the second mass spectrometry (MS/MS) analysis (Mass Spectrometry data S1), the result revealed 4 matched peptides with 31% sequence coverage. The matched peaks were zoomed in, and the sequences are shown in red. The Probability Based Mowse Score was 160 (>56, p < 0.05) (Table S1). (**B**) mRNA levels of eIF5A1 were measured using qPCR where β-actin was an internal control. (**C**) Protein levels of eIF5A1 were measured using WB, and normalized to β-actin; quantitation was done using Image J. (**D**) Expression of eIF5A1 (red) was shown to localize to neurons (NeuN^+^) (green). Nuclei were visualized by DAPI (blue). Bar, 30 μm. Data were presented as mean ± SEM. *P < 0.05 compared with the sham group; **P < 0.01 compared with the sham group; ^#^P < 0.05 compared with the 14 dpo group.

Amino acid sequences from four peptides in spot 11 (Fig. 1B) constituted 31 percent of the eIF5A1 amino acid sequence. The observed PI and molecular mass of the isolated protein were 5.08 and 16821Da whereas the calculated PI and molecular mass were 4.76 and 22300 Da. The Probability Based Mowse Score was 160 where a score greater than 56 was considered significant (additional details about the mass spectrometry data are described in supplemental mass spectrum data S1 and Table S1). We went on to confirm changes in the expression of eIF5A1 within the spinal cord after SCT using quantitative PCR (qPCR) and western blotting (WB). The mRNA levels of eIF5A1 were significantly upregulated in SCT rats 28 dpo compared to the SCT rats 14 dpo (p < 0.05) (Fig. 2B). Moreover, eIF5A1 protein levels were significantly increased in SCT rats 28dpo compared to SCT rats 14 dpo (p < 0.05) (Fig. 2C). Within the spinal cord caudal to the injury, eIF5A1 was expressed mainly in neurons of the spinal cord (Fig. 2D).

### eIF5A1 improves functional recovery *in vivo* possibly by upregulating neurofilament expression and promoting neuronal survival.

**Figure 3:**
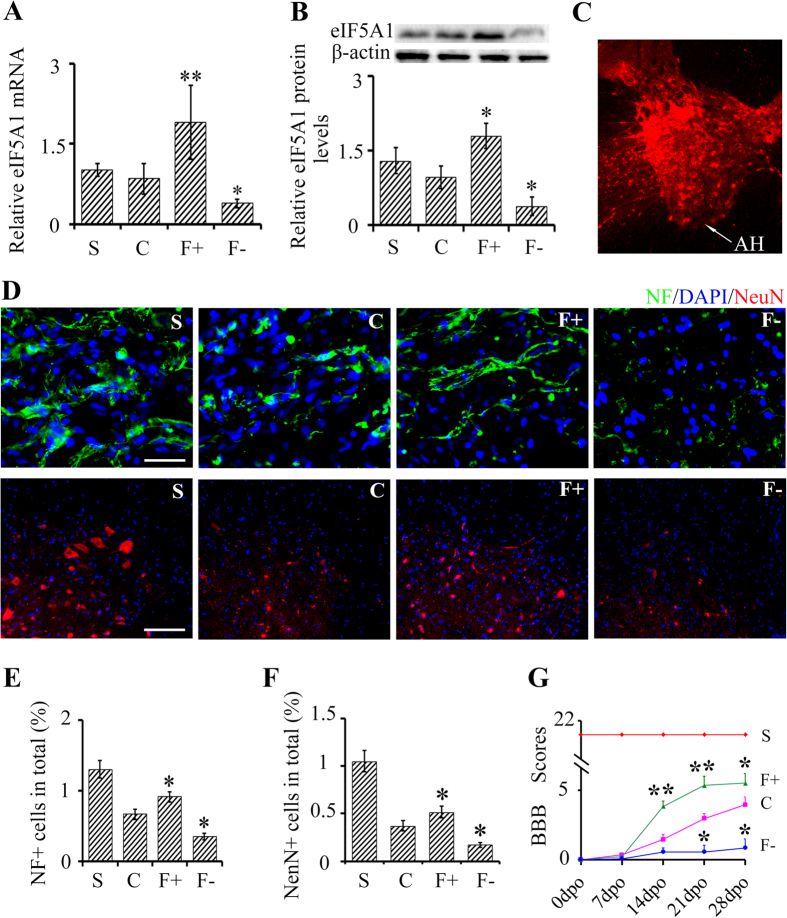
Overexpression of eIF5A1 upregulates neurofilament expression, promotes neuronal survival and improves functional recovery *in vivo.* (**A,B**) eIF5A mRNA (**A**) and protein levels (**B**) were measured 28 days after lentiviral injections (described below) and SCT with β-actin as an internal control. (**C**) Expression of control lentiviral constructs in the anterior horn (AH) cells of the spinal cord. (**D**) Levels of neurofilament (NF+) (green) and NeuN^+^ neurons (red) were measured 28 days after injection of lentiviral constructs caudal to SCT. Nuclei were visualized by DAPI (blue). Bar (NeuN^+^), 100 μm. Bar (NF+), 30 μm. Quantifications are shown in part panels (**E–G**) BBB scores were used to assay hind limb motor function. The uninjured sham controls rats had BBB scores of 21 ± 0.00. BBB scores of rats overexpressing eIF5A1 were higher than control rats while rats with reduced eIF5A1 had lower BBB scores. S, sham group; C, SCT, control lentiviral construct; F+, SCT, eIF5A1 overexpressing lentiviral construct; F–, SCT, shRNA-eIF5A1 expressing lentiviral construct; Data were presented as mean ± SEM. *P < 0.05 compared with the control group; **p < 0.01 compared with the control group.

Having confirmed that eIF5A1 is markedly upregulated after SCT, we wanted to determine whether the targeting of eIF5A1 *in vivo* is an effective mechanism for neuroplasticity. To accomplish this, we generated lentivirual constructs to overexpress eIF5A1; vector information is shown in Fig. S1A–C. The sequence for the eIF5A1 shRNA vector was obtained from a previous study^17^. Additional vector information and expression of the appropriate reporter genes are shown in Fig. S1D,E. Initially, to confirm expression from our lentiviral constructs, we injected a control lentiviral RFP expressing construct in spinal cords of uninjured rats. RFP reporter was expressed in anterior horn (AH) cells that were transduced by the control lentiviral construct (Fig. 3C). To increasing the transduction efficiency, we decided to reduce the size of the lentiviral construct used to modify expression levels of eIF5A1 or RhoGDIα in the subsequent experiments by removing the reporter genes within the lentiviral vectors.

Next, we injected lentiviral constructs caudal to T8 and induced SCT at T8. We confirmed the changes of eIF5A1 in the spinal cord caudal to the injury where the levels of eIF5A1 mRNA was upregulated (p < 0.01) in eIF5A1 overexpression group, and downregulated (p < 0.05) in eIF5A1 interference group compared to the negative control group (Fig. 3A). Correspondingly to the mRNA data, eIF5A1 protein levels increased in the overexpression group (p < 0.05) and decreased in the interference group (p < 0.05) (Fig. 3B). In addition, levels of neurofilament (NF), a regeneration-associated protein, correlated with expression levels of eIF5A1; NF increased in the overexpression group (p < 0.05) and decreased in the interference group (p < 0.05) (Fig. 3D,E). Similarly, number of neurons (NeuN^+^) correlated with expression levels of eIF5A1 (p < 0.05) (Fig. 3D,F). Furthermore, overexpression of eIF5A1 improved motor function after SCT as measured by BBB scores compared to control lentiviral construct whereas a decrease of eIF5A1 reduced motor function (Fig. 3G). Collectively, this data shows that eIF5A1 expression is associated with CNS axonal regeneration.

### eIF5A1 regulates neuronal survival and axon growth *in vitro*

**Figure 4:**
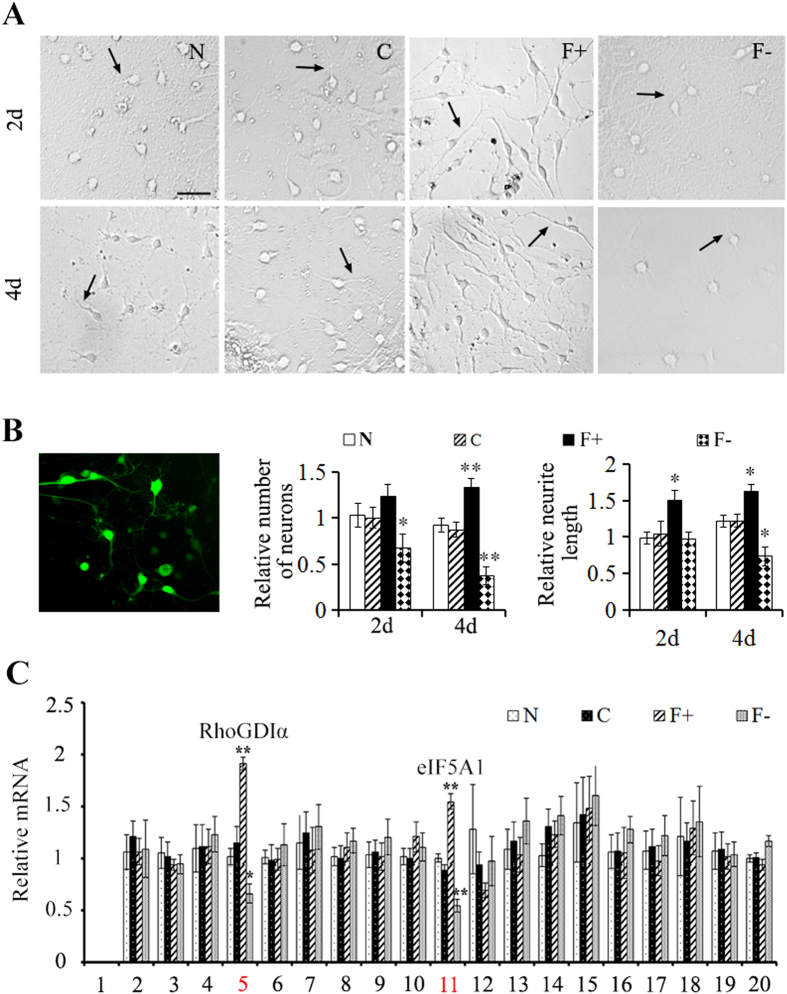
Overexpression of eIF5A1 promotes neuronal survival and axonal growth *in vitro*. (**A**) Primary neurons were cultured for 2 and 4 days (d) after transduction with lentiviral construct described in Fig. 3. Bar, 50 μm. (**B**) Expression of control lentiviral construct (green) in cultured primary cord neurons. Numbers of neurons and neurite length from panel A were quantified by Leica LAS AF software. And all groups were normalized to normal (N) group at 2d. (**C**) After transduction of lentiviral construct described in Fig. 3 in cultured primary neurons, mRNA level of 20 proteins identified in Fig. 1 were quantified using qPCR. The RhoGDIα (spot 5) was significantly upregulated in eIF5A1 overexpressed group, while it was significantly downregulated in eIF5A1 reduced group. Albumin (spot 1) was not detected. Data were presented as mean ± SEM. *P < 0.05 compared with the control group; **p < 0.01 compared with the control group.

Given the role of eIF5A1 on neuroplasticity *in vivo*, we next aimed to study the effects of eIF5A1 in primary neurons. Toward this we transduced primary neurons with lentiviral overexpressing eIF5A1 and shRNA to inhibit eIF5A1 (Fig. 4A,B left panel). Primary neurons overexpressing eIF5A1 exhibited a significant increased in cell number and neurite length compared to the control group (p < 0.05) whereas neurons with reduced levels of eIF5A1 exhibited a decrease in the number of neurons and neurite length (p < 0.05) (Fig. 4A,B middle and right panels). These results showed that eIF5A1 promotes cell survival and to neurite outgrowth.

### eIF5A1 regulates RhoGDIα expression in cultured primary neurons

**Figure 5:**
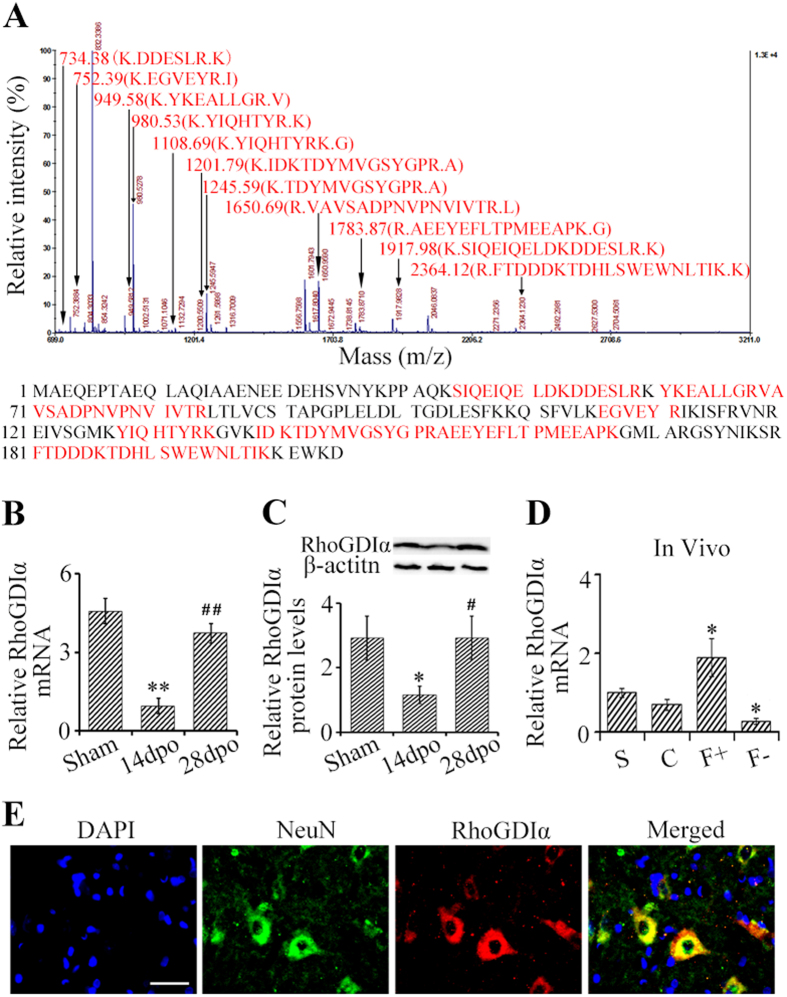
RhoGDIα was upregulated in the spinal cord, caudal to the SCT from 14 dpo to 28 dpo. (**A**) Primary mass spectrometry result of spot 5 (RhoGDIα). After the mass spectrometry (MS/MS) analysis (Mass Spectrometry data S2), the result revealed 11 matched peptides with 50% sequence coverage. The matched sequences are shown in red. The Probability Based Mowse Score was 237 (>56, P < 0.05) (Table S1). (**B**) mRNA levels of RhoGDIα were measured using qPCR where β-actin was an internal control. (**C**) Protein levels of RhoGDIα were measured using WB, and normalized to β-actin; quantitation was done using Image J. (**D**) mRNA levels of RhoGDIα from the uninjured spinal cord (T8) were assayed following injection of lentiviral constructs that modify expression eIF5A1 (these constructs are described in Fig. 3; β-actin was an internal control. (**E**) Expression of RhoGDIα (red) was shown to localize to neurons (NeuN^+^) (green). Nuclei were visualized by DAPI (blue). Bar, 30 μm. Data were presented as mean ± SEM. *P < 0.05 compared with the sham or control group; **P < 0.01 compared with the sham group; ^#^P < 0.05 compared with the 14 dpo group; ^##^P < 0.01 compared with the 14 dpo group.

As previously stated, we identified 20 proteins that are differentially expressed in the spinal cord caudal to the injury during spontaneous functional recovery after SCT. Up to this point, we determined that one of these proteins, eIF5A1 does indeed play a role in functional recovery after SCT. Since eIF5A1 regulates protein synthesis, we aimed to determine what are targets that eIF5A1 regulates. Toward this, we overexpressed and inhibited eIF5A1 in primary neurons, and measured mRNA levels of the other 19 proteins. Levels of RhoGDIα (spot 5) mRNA increased in primary neurons overexpressing eIF5A1 (p < 0.05), and decreased in neurons with decreased levels of eIF5A1 (p < 0.05) (Fig. 5D).

### Validation of expression of RhoGDIα after SCT

Amino acid sequences from eleven peptides in spot 5 (Fig. 5A) constituted 50 percent of the RhoGDIα amino acid sequence. The observed PI and molecular mass of the isolated protein were 5.12 and 23393 Da whereas the calculated PI and molecular mass were 5.38 and 21820 Da. The Probability Based Mowse Score was 237 where a score greater than 56 was considered significant (additional details about the mass spectrometry data are described in supplemental mass spectrum data S2 and Table S1). Again, we went on to confirm changes in the expression of RhoGDIα within the spinal cord after SCT using quantitative PCR (qPCR) and western blotting (WB). The mRNA levels of RhoGDIα were significantly upregulated in SCT rats 28 dpo compared to the SCT rats 14 dpo (p < 0.05) (Fig. 5B). Similarly, RhoGDIα protein levels were significantly increased in SCT rats 28 dpo compared to SCT rats 14 dpo (p < 0.05) (Fig. 5C). In addition, mRNA levels of RhoGDIα were upregulated in rats overexpressing eIF5A1 (p < 0.05), and were downregulated in rats with decreased eIF5A1 (p < 0.05) (Fig. 5D). RhoGDIα was also expressed mainly in spinal cord neurons caudal to the injury (Fig. 5E).

### eIF5A1 upregulates RhoGDIα to promotes neuroplasticity and functional recovery after SCT

**Figure 6:**
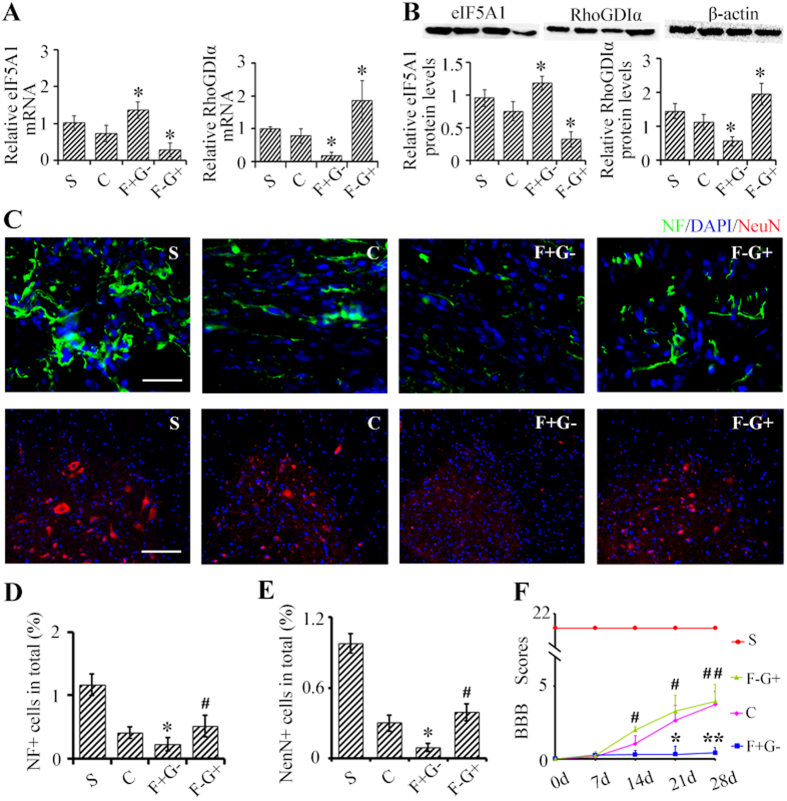
eIF5A1 upregulates RhoGDIα to promote neuroplasticity caudal to the SCT and functional recovery after SCT. (**A,B**) eIF5A1 and RhoGDIα mRNA (**A**) and protein levels (**B**) were measured 28 days after lentiviral injections (described below) and SCT with β-actin as an internal control. (**C**) Levels of NF (green) and number of NeuN^+^ neurons (red) were measured 28 days after injection of lentiviral constructs caudal to SCT. Nuclei were visualized by DAPI (blue). Bar (NeuN^+^), 100 μm. Bar (NF +), 30 μm. Quantifications are shown in part panels (**D–F**) BBB scores were used to assay hind limb motor function. The uninjured sham controls rats had BBB scores of 21 ± 0.00. BBB scores of rats in group F + G- where lower than the control but recovered in the F-G + group. Indeed, BBB scores were statistically indistinguishable to the control group. S, sham group; C, SCT, control lentiviral construct; F + G-, SCT, eIF5A1 overexpressing and shRNA-RhoGDIα expressing lentiviral constructs; F-G + , SCT, shRNA-eIF5A1 expressing and RhoGDIα overexpressing lentiviral constructs. Data were presented as mean ± SEM. *P < 0.05 compared with control group; **P < 0.01 compared with control group; ^#^P < 0.05 compared with the F + G- group; ^##^P < 0.01 compared with the F + G- group.

To determine eIF5A1 and RhoGDIα, we first generated lentivirual constructs to overexpress RhoGDIα; vector information is shown in Fig. S2A–C); and, then we generated a lentiviral construct that inhibited expression of RhoGDIα by expressing a RhoGDIα shRNA (Fig. S2D–F). Next, we injected a combination of lentiviral constructs that overexpress eIF5A1 and inhibit RhoGDIα (F + G-) or lentiviral constructs that inhibit eIF5A1 and overexpresses RhoGDIα (F-G + ) caudal to T8 level in the spinal cords to determine if we can alter levels of eIF5A1 and RhoGDIα when transducing spinal cord neurons with a combination of lentiviral constructs. Compared with control rats (transduced with empty lentiviral constructs), the F + G- group showed a significant upregulation of eIF5A1 mRNA and downregulation of RhoGDIα mRNA (p < 0.05). In turn, the mRNA level of eIF5A1 was markedly downregulated in the F-G + group while RhoGDIα mRNA was upregulated compared to the control rats (p < 0.05) (Fig. 6A). Protein levels mirrored these results (p < 0.05) (Fig. 6B).

To determine whether eIF5A1 and RhoGDIα are in the same pathway, rescue experiments were carried out where we injected F + G- and F-G + lentiviral constructs caudal to T8 and induced SCT at T8; experimental endpoint was 28d after SCT. In the F + G- group, the downregulation of RhoGDIα resulted in reduced levels of NF (p < 0.05) whereas in the F-G + group, overexpression of RhoGDIα rescued levels of NF (p < 0.05) despite the reduced levels of eIF5A1 (Fig. 6C,E). Similarly, number of neurons (NeuN^+^) was reduced in the F + G- group (p < 0.05) but was rescued in the F-G + group (p < 0.05) (Fig. 6C,E). Notably, the rescue effect of RhoGDIα overexpression extended to motor function. Through 0, 7, 14, 21 and 28 dpo time points, BBB scores were reduced in the F + G- group compared to the control group (p < 0.05) but recovered to control levels in the F-G + group (p < 0.05) (Fig. 6F). Collectively, our data indicates that eIF5A1 and RhoGDIα are in the same signaling pathway where eIF5A1 modulates RhoGDIα. Indeed, upregulation of RhoGDIα through this pathway *in vivo* promotes neural regeneration that generates the spontaneous recovery of motor function following SCT.

### eIF5A1 upregulates RhoGDIα in neurons to promote neuronal regeneration *in vitro*

**Figure 7:**
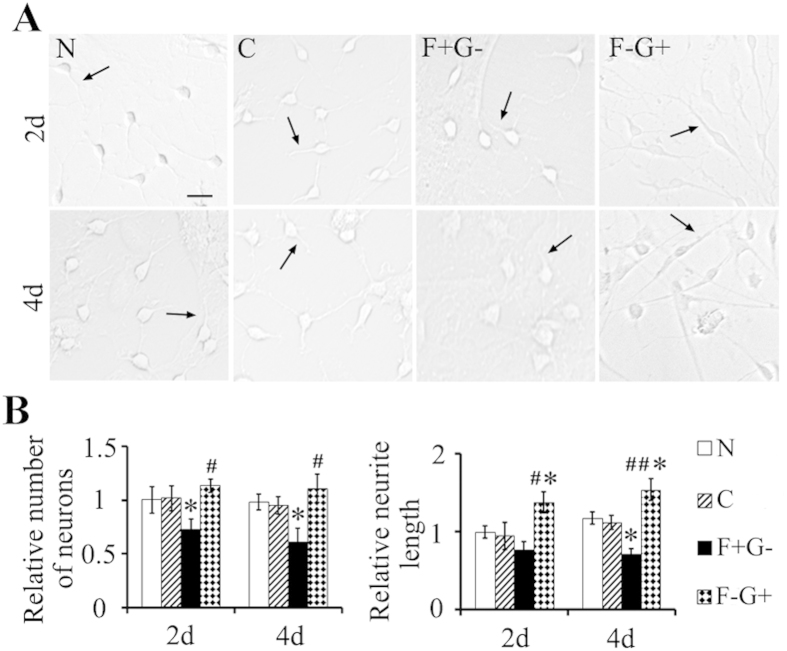
eIF5A1 upregulates RhoGDIα in neurons to promote neuronal survival and axonal growth *in vitro.* (**A**) Primary neurons were cultured for 2 and 4 d after transduction with lentiviral construct described in Fig. 6. Bar, 50 μm. (**B**) Numbers of neurons and neurite length from panel A were quantified by Leica LAS AF software. And all groups were normalized to normal (N) group at 2d. Data were presented as mean ± SEM. *P < 0.05 compared with control group; ^#^P < 0.05 compared with the F + G- group; ^##^P < 0.01 compared with the F + G- group.

**Figure 8:**
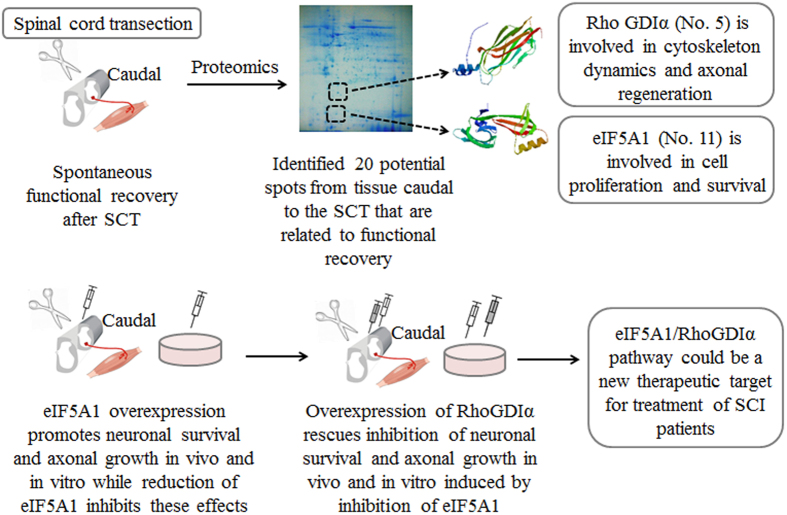
eIF5A1/RhoGDIα pathway: a novel therapeutic target for treatment of spinal cord injury was identified using a proteomics approach. The restoration of neurological functions after CNS injury is limited. Such limited functional recovery is observed in rodents and primates as well^7^,^34^. Here, we seek to identify possible mechanisms through which such spontaneous function recovery occurs, and to explore the new potential therapeutic targets for SCI and other CNS injuries. Using a proteomics approach we detected 20 proteins expressed differentially in the spinal cord caudal the epicenter of the injury. Of these proteins, we focus on eIF5A1 and RhoGDIα^41^,^42^. eIF5A1 is a protein with function in cell proliferation and survival while RhoGDIα is involved in cytoskeleton dynamics and promotes axonal growth. Lentiviral vectors were used to manipulate their expression *in vivo* and *in vitro* in order to study their roles in neuroplasticity and functional recovery after SCT. Consequently, we found that eIF5A1 regulates RhoGDIα to promote neuroplasticity and functional recovery. This pathway might play a pivotal role in enhancing the level of functional recovery and in turn improve patients’ quality of life. Finally, Fig. 8 was drawn by one of our co-authors, Dr. Fei-Fei Shang.

To confirm that the regenerative eIF5A1/RhoGDIα mechanism functions in neurons, we performed rescue experiment *in vitro* using cultured primary neurons (Fig. 7). We used the same combinations of lentiviral constructs to transduce primary spinal cord neurons, and examined the number of neurons and neurite lengths 2 and 4 d after lentiviral transduction (Fig. 7A). The reduction RhoGDIα in the F + G- group resulted in reduced numbers of neurons in both time points (p < 0.05), and reduced neurite lengths but only 4d after transduction (p < 0.05). Overexpression of RhoGDIα in the F-G + group rescued neurons in both time points (p < 0.05), and increased neurite lengths compared to the control groups (p < 0.01) (Fig. 7B). This data demonstrated that eIF5A1/RhoGDIα pathway promotes neuronal survival and axonal regeneration in neurons. A summed schematic model was showed at Fig. 8.

## Discussion

Axonal damage at the epicenter of pathologies such as stroke, traumatic brain injury (TBI) and SCI patients can result in signal conduction failure at the epicenter of their pathology due to which in turn causes persistent functional deficit. Restoration of neurological functions or neuroplasticity does occur in these CNS pathologies but it is limited^1^,^3^,^12^,^13^,^17^. A potential mechanism to induce neuroplasticity may be involved creating remodeling the local circuitry in the spinal cord^6^,^13^,^17^,^28^. We hypothesize that increasing neuronal survival and inducing axonal regeneration will enhance neuroplasticity and will contribute to functional recovery. In this study, using 2D electrophoresis and mass spectrometry, we found 20 proteins expressed caudal to a SCT that are potentially involved in spontaneous functional recovery. These proteins were sorted into groups based on their known functions including proliferation and apoptosis, transport, differentiation, cytoskeleton, metabolism, translation, cell signaling, protein folding, and other functions.

One protein that was upregulated following SCT is eIF5A1, a protein involved in cell proliferation and survival^29^,^30^,^31^. eIF5A1 is an isoform of the eIF5A gene that shares 84 percent sequence homology and 92 percent sequence similarity with its other homologue, eIF5A2. Both homologes contain the unusual amino acid, hypusine, which is reduced by 75% following exposure to the cytokine IFNa but recovers following a 12 h exposure to EGF^15^,^29^. The hypusine is believed to be important to a common function of these homologues because the sequence around this amino acid is highly conserved^29^. However, their expression patters are very different with eIF5A2 being expressed in testes (high levels), brain (low levels), and cancer cells^29^,^32^. On the other hand, eIF5A1 is expressed ubiquitously in mouse embryos by E13.5. Particularly tissues undergoing differentiation have higher levels of eIF5A1^16^,^30^,^31^. In addition to differentiation, eIF5A1 stimulates the proliferation and survival of various cells types such as multipotent adult germline stem cells, embryonic stem cells and corneal epithelial cells^15^,^31^. However, eIF5A1 has been shown to facilitate translation elongation predominantly in adverse environments^30^,^31^. In addition, eIF5A1 can act to initiate translation of protein synthesis as an important partner of eIF2^30^. Here we report that eIF5A1 enhances neuroplasticity and functional recovery after SCT. Furthermore, we show that eIF5A1 mediates these effects by upregulating RhoGDIα. Therefore, while the role of eIF5A1 described in this work fits in with previously described functions of eIF5A1 (increased cell survival and modification of protein levels), the target of eIF5A1, RhoGDIα, and the effect, enhanced neuroplasticity and functional recovery, are completely novel. This role of eIF5A1 does not appear to be mediated by hypusine amino acid and does not appear to be shared with eIF5A2 because downregulation of eIF5A1 alone reduces neuroplasticity and functional recovery after SCT.

The target of eIF5A1, RhoGDIα, is a member of GDI family of proteins that plays a role in cytoskeletal dynamic. The GDP dissociation inhibitors (GDIs) are pivotal regulators of Rho GTPases, which are essential in tumor progression particularly in the area of metastasis^24^,^26^,^27^. The mammalian Rho GTPase family consists of at least 14 different members: Rho (A, B, and C), Rac (1, 2, and 3), Cdc42, RhoD, RhoG, RhoH/TTF, TC10, and Rnd (1, 2, and 3). These proteins act as intracellular molecular switches that transduce extracellular signals to the actin cytoskeleton. Rho GTPases are also involved in neuronal morphogenesis, axon growth and guidance, dendrite development, plasticity, and synapse formation. The best-characterized family members are RhoA, Rac1, and Cdc42, each belonging to a different subgroup of the Rho family, the RhoA, Cdc42, and Rac subfamilies, respectively^19^,^21^,^25^,^26^. RhoA is known to block axonal regeneration and participate in apoptosis after SCI. RhoA activation after SCI is also involved in oligodendrocyte apoptosis via p75 neurotrophin receptor activation^18^,^23^,^25^,^33^. Inhibition of the Rho-associated kinase (ROCK), a downstream target of RhoA, has been shown to promote axon regeneration and to improve functional recovery following SCI^10^,^11^,^34^. In the CNS, RhoGDIα could stimulate RhoA to regulate cell growth, neuronal differentiation and axonogenesis^21^. Functions of RhoGDIα have been previously described but our finding that eIF5A1 induces RhoGDIα and increases neuroplasticity and functional recovery in the spinal cord flowing SCT has not been described.

Several other proteins related to spontaneous functional recovery were also identified through our proteomics approach including glial fibrillary acidic protein (GFAP), vimentin, transferrin, glyceraldehyde-3-phosphate dehydrogenase (GAPDH), and fatty acid binding protein (FABP).

GFAP and vimentin are overexpressed in reactive astrocytes that are found in glial scar that surround the epicenter of traumatic CNS injuries and inhibit axonal regeneration^33^. Vimentin is one of the most widely expressed mammalian intermediate filament proteins, and in addition to reactive astrocytes it is expressed in fibroblast and differentiating cells. Double knockout mice for GFAP and vimentin have reduced astrocyte reactivity and increased axonal regeneration after spinal cord and brain injuries^1^,^35^. Additionally, microRNA-138 downregulates vimentin to reduce astrogliosis and to promote motor functional recovery in SCT rats^36^.

Iron overload results in oxidative stress; therefore, intracellular iron load is tightly controlled via the transferrin, transferrin receptor (TfR) and ferritins. Transferrin carries iron throughout the body, minimizing the amount of free iron thus reducing free radical formation^11^. Subcortical astrocytes have been shown to contain transferrin with increasing age. This supports the hypothesis that following trauma, increased levels of transferrin will protect against iron mediated free radical damage^3^,^5^,^12^. Therefore, regulation of these proteins may be critical for neuroprotection^6^,^10^.

GAPDH plays a role in glycolysis and nuclear functions. It catalyzes an important energy-yielding step in carbohydrate metabolism, the reversible oxidative phosphorylation of glyceraldehyde-3-phosphate in the presence of inorganic phosphate and nicotinamide adenine dinucleotide^34^. GAPDH participates in nuclear events including transcription, RNA transport, DNA replication and apoptosis, and modulates the organization and assembly of the cytoskeleton. In addition, GAPDH facilitates CHP1-dependent microtubule and membrane associations through its ability to stimulate the binding of CHP1 to microtubules^37^. The protein was decreased in SCI, and it also determined presented with methionine loss, phenylalanine oxidation at F45, and proline oxidation at P127 that would confirm oxidative stress in spinal cord at this time point^35^,^38^.

FABP binds hydrophobic ligands and is involved in the intracellular transport of these molecules through the cytoplasm and cell nucleus^1^. It has previously been demonstrated that hindlimb unloading in rats downregualtes FABP whereas in human exercise upregulates FABP expression^11^. Release of free fatty acids (FFAs) is increased in SCI where it contributes to edema and inflammation related to secondary tissue damage^8^. An increase in FABP may be a protective response to the increase in FFA after SCI^39^. Also it suggests that cells in the injured spinal cord may mobilize FFAs to synthesize new membranes^4^.

In this study, we conclude that eIF5A1 and RhoGDIα play a role in the spontaneous functional recovery after SCI. Considering that these two proteins are part of the same signaling pathway, we believe we have identified a new target for clinical intervention with the goal of increasing functional recovery in patients with SCI and other CNS injuries such as TBI.

## Methods

### Animals

Animal care and all experimental protocols were approved by the Sichuan University Committee on Animal Research, and consistent with the ethical guidelines of the National Institutes of Health^40^. Adult female Sprague-Dawley (SD) rats (Dashuo, China) weighing 250–300 g were used for all behavioral and biochemical studies. National Institutes of Health (NIH) guidelines for laboratory animal care and safety have been followed. Animals were housed in 25 °C ± 1.0 °C, kept in groups of four and maintained on a 12 h light/dark cycle; food and water were given ad libitum.

### Surgery and Lentivirus injection

In order to reduce the size of the lentiviral constructs for increased transfection efficiency, the lentiviral constructs used to regulate expression of eIF5A1 or RhoGDIα didn’t contain a fluorescent reporter gene. Surgical procedures were performed as described previously^17^. Briefly, a total of 10 μl lentivirus (0.2 μl/min) was injected into spinal cord, caudal to the injury, via a glass micropipette; 2.5 μl were injected in four spots. The empty vector was injected as control. Then, the spinal cord was transected at level T8. Throughout surgery and during post-operative recovery period, animals were maintained at normal body temperature with a feedback-controlled heating blanket. The animal groupings were list in Table S3, Table S4 and Table S5 (Supplementary Information).

### Evaluation of motor function

BBB locomotor rating scale was used to assess motor function after SCT (Basso *et al*., 1995). A score of 0 registers complete paralysis and a score of 21 represents complete mobility. Blind scoring ensured that observers were not aware of the treatments received by each rat.

### Proteomics analysis

As described previously^17^, we extracted protein from 0.5 cm caudal cord tissue to the injury with lysis buffer (7M urea, 2Mthiourea, 4% CHAPS, 100 mM DTT, 0.2% pH3-10 ampholyte, Bio-Rad, USA) containing protease inhibitor (Sigma). Total protein (1.2mg) was loaded into IPG strips (17 cm, pH3-10, Bio-Rad), and separated by isoelectric focusing (first dimension) and 12% SDS-PAGE (second dimension); the gels were stained with CBB G-250. Quantitation and comparison of each gel spot was done with PDQuest software 7.4 (Bio-Rad). Only those spots that changed consistently and significantly (more than 1.5-fold) were selected for TOF/TOFTM analysis. To ensure the consistency of the data, each of the paired samples was run twice for 2-D analysis.

The gel spots were de-stained twice with 0.1 ml of 50 mM NH_4_HCO_3_, and 50% acetonitrile for 20 min and dehydrated in 100% acetonitrile for 10 min. Protein digestion was carried out with mass spectrometry grade trypsin (Trypsin Gold, Promega). Samples were analyzed on a 4700 proteomics analyzer (Applied Biosystems, Foster City, CA, USA). The generated MS and MS/MS spectra were subsequently submitted to MASCOT (version 2.1, Matrix Science, London, UK) by GPS Explorer software (version 3.6, Applied Biosystems). The individual MS/MS spectrum with a statistically significant (confidence interval ≥ 95%) ion score was accepted. Only protein scores that are greater than 56 (p < 0.05) were reported.

### Quantitative PCR (qPCR)

Total RNA was extracted from spinal cord tissue caudal to the injury using TRIzol reagent (Life Technologies) according to the manufacturer’s protocol and reversed transcription reaction was performed with the RevertAid^TM^ First Strand cDNA Synthesis kit (Thermo). PCR was performed in a DNA thermal cycler (CFX 96, Bio-Rad) according to the following standard protocol: one cycle of 95 °C for 2 min; 40 cycles of 95 °C for 10 s, annealing for 15 s and 72 °C for 30 s. Relative expression levels were calculated with normalization to β-actin values by using the 2^–△△Ct^ method. The primer sequences are listed in Table S2.

### Western blotting

Frozen spinal cord tissue samples (200 mg) isolated caudal to the injury were crushed in liquid nitrogen. Total protein extracts (100 ug/lane) were resolved using 10 or 15% SDS-PAGE and analyzed by western blot as described previously^17^. All samples were incubated overnight at 4 °C with the following primary antibodies, rabbit anti-eIF5A1, 1:2000, Abcam; rabbit anti-RhoGDIα, 1:1000, Abcam; rabbit anti-β-actin, 1:5000, Abcam. Horseradish peroxidase-coupled secondary antibodies (1:5000; Abcam) were incubated in TBST for 1 h at room temperature. All samples were visualized using ECL detection reagents (Beyotime, China). Quantitative densitometric analysis was done using Image J software.

### Immunofluorescence staining

Section preparation and staining were carried out as described previously^17^. Briefly, after the final BBB evaluation, animals were perfused with 4% paraformaldehyde solution. Spinal cord tissue (0.5 cm caudal to the injury) was harvested and placed in 4% paraformaldehyde then successively in 0.1 M phosphate buffered saline solutions that contained 10%, 20% and 30% sucrose. Spinal cord tissue was sectioned at 20 μm thickness in a freezing microtome (Leica CM1900, Germany). Every 50 sections, 1 slide was selected for immunofluorescence staining. After blocking for 1 hr with 10% normal goat serum, sections were incubated in primary antibody (eIF5A1, 1:200, Abcam; RhoGDIα, 1:200, Abcam; SMI-312(NF), 1:500, Covance; NeuN, 1:400, Abcam). Goat anti-rabbit (1:400, Invitrogen) and goat anti-mouse (1:400, Invitrogen) were used as secondary antibody. Lastly, sections were imaged with Leica AF6000 fluorescence microscope. The neurofilament positive cells (NF+) and NeuN positive cells (NeuN+) were measured by Leica LAS AF software. We used this software to compute the positive cells in spinal gray matter per mm^2^. The computing method of Fig. 3E,F and Fig. 6 D,E is following. Positive cells (NF+ or NeuN+) in total (%) = (positive cells number/total cells number (DAPI + nucleus)/area (mm^2^))*100.

### Lentivirus package

eIF5A1 (NM_001033681.1) and RhoGDIα (NM_001007005.1) sequences were obtained from NCBI. Interference mRNA sequence of eIF5A1 (GCAAGGAGATTGAGCAGAA) was obtained from a previous study^17^. The most effective interference sequence for RhoGDIα (named fragment 2) was identified in PC12 cells. The sequence for fragment 2 is GGAACTGGACAAGGATGAT.

As described previously^17^, the lentiviral packaging was done according to HIV Expression Packaging Kit User Manual of GeneCopoeia. Briefly, vector transfection was performed in 70–80% confluent 293T cells; 12 h later, the Titer Boost reagents were added. At 48h post transfection, the culture medium was collected, centrifuged, and then the supernatant was filtered. Lentiviral samples were stored at −80 °C until use.

### Primary culture of spinal cord neurons and lentivirals transductions

Spinal cords of neonatal rats were used to isolate primary neurons. Primary neurons were cultured as described previously with slight modifications^17^. Spinal cords were dissected and digested in 0.3% trypsin for 30 min at 37 °C. After addition of DMEM cultured medium supplemented with 10%BSA, spinal cord tissue was centrifuged at 1000 g for 10min, and then the supernatant was discarded. Cells were plated on culture dishes in Neurobasal medium (Gibco) supplemented with B27 (Gibco), and cultured for 2 weeks. Spinal cord primary neurons were transduced with lentivirus using 1% polybrene (Sigma) according to manufacturer’s protocol. Primary neuron cultures were imaged 2d and 4d after transduction using Leica AF6000 fluorescence microscope; each experiment was repeated 3 times. Each well was taken 5 pictures (200 ×) that were in above, below, left, right and middle of the well. Cell number and neurite length were measured by Leica LAS AF software.

### Statistical analysis

Data is expressed as means ± standard error of mean (SEM) and was analyzed using SPSS 13.0. Comparisons between two groups were made with unpaired Student’s t-tests. Non-parametric comparisons between three or more groups were made with a one-way ANOVA test. In all cases, p < 0.05 was considered statistically significant.

## Additional Information

**How to cite this article**: Liu, W. *et al.* eIF5A1/RhoGDIα pathway: a novel therapeutic target for treatment of spinal cord injury identified by a proteomics approach. *Sci. Rep.* **5**, 16911; doi: 10.1038/srep16911 (2015).

## Supplementary Material

Supplementary Information
