# Supplementary material for: eIF5A1/RhoGDIα pathway: a novel therapeutic target for treatment of spinal cord injury identified by a proteomics approach
Source: Sci Rep. 2015 Nov 23;5:16911. doi: 10.1038/srep16911 (PMC4655360; doi:10.1038/srep16911)
Supplement: Supplementary Information [file srep16911-s1.pdf]

eIF5A1/ RhoGDIα pathway: a novel therapeutic target for treatment of spinal cord injury identified by a proteomics approach

Supplementary Information

Wei Liu[1#], Fei-Fei Shang[1#], Yang Xu[1], Visar Belegu[4], Lei Xia[1], Wei Zhao[1], Ran Liu[1], Wei Wang[1], Jin Liu[1], Chen-Yun Li[3*],Ting-Hua Wang[1,2*]

# These authors contributed equally to this work.

[1]Department of Anesthesiology and Translational Neuroscience Center, the State Key Laboratory of Biotherapy, West China Hospital, Sichuan University, Chengdu 610041, PR China, [2]Institute of Neuroscience, Kunming medical University, Kunming 650031, PR China, [3]Key Laboratory of Agro-Biodiversity and Pest Management of Education Ministry of China, Yunnan Agricultural University, Kunming, 650000, P.R. China, [4]Department of Neurology, Johns Hopkins School of Medicine, Baltimore, MD, USA

*Correspondence to: Ting-Hua Wang, Department of Anesthesiology and Translational Neuroscience Center, West China Hospital, Institute of Neurological Disease, the State Key Laboratory of Biotherapy, Sichuan University, P.R. China. Tel: +86-28-85501036; Fax: +86-28-85501036; E-mail: tinghua_neuron@263.net or Key Laboratory of Agro-Biodiversity and Pest Management of Education Ministry of China, Yunnan Agricultural University, Kunming, Yunnan, P.R. China, E-mail: li.chengyun@gmail.com.

Supplemental Figures

Figure S1

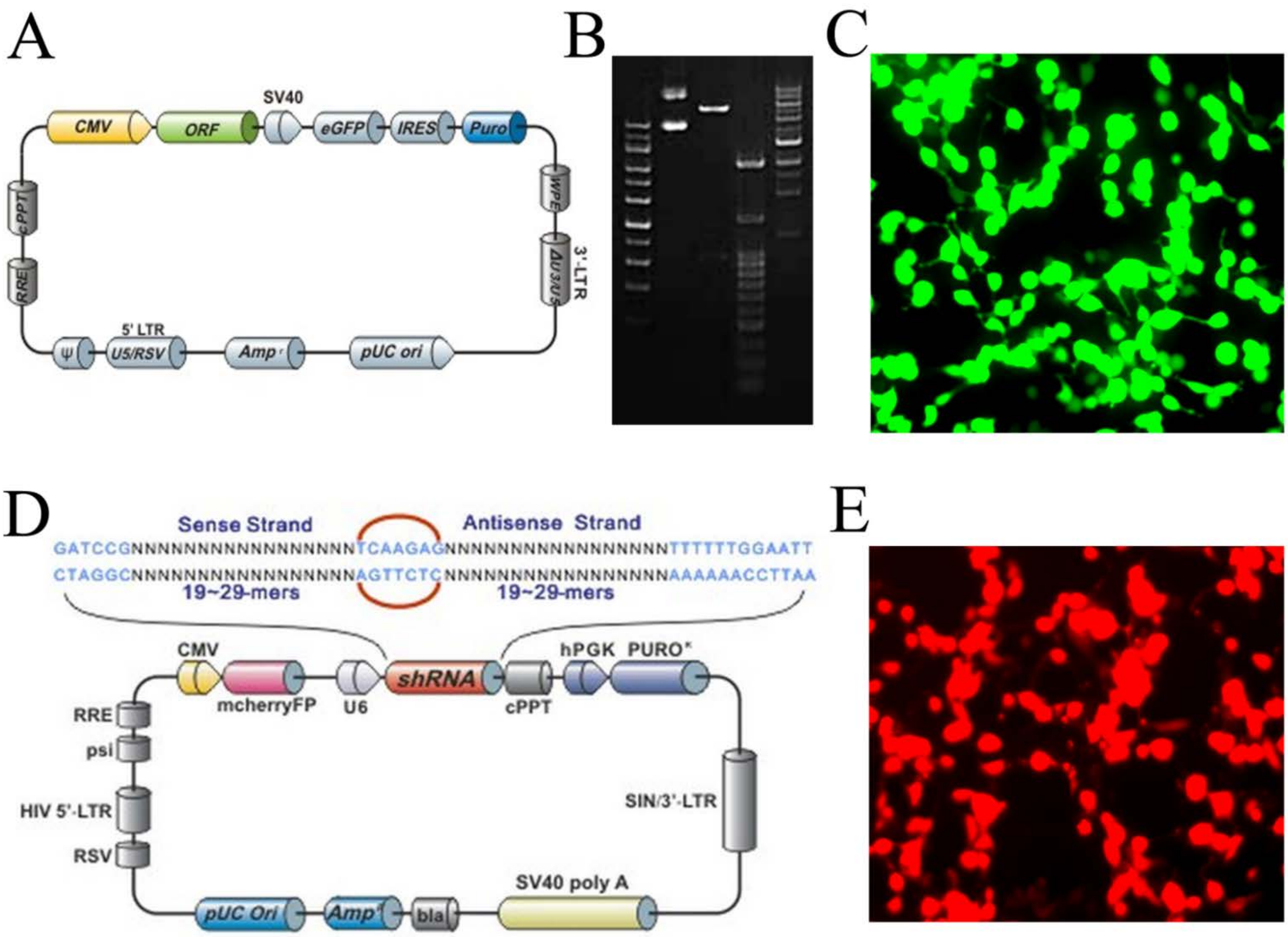

Figure S1. Packaging of recombinant eIF5A1 Lentivirus

(A) Vector information of ORF sequence for EX-Rn10147-Lv201. Puromycin and ampicillin are stable selection markers that can be used for selecting stably transduced cells. (B) Verify the correctness of Clone EX-Rn10147-Lv201. EX-Rn10147-Lv201 was digested by EcoRI and XhoI restriction enzyme and run on 1.0% agarose gel. Lane1: DNA Ladder 6000. Lane2: EX-Rn10147-Lv201 plasmid. Lane3: EX-Rn10147-Lv201 plasmid digested. There are two expected bands (~1074/7963bp). Lane4: DNA Ladder 3000. Lane5: DNA Ladder 15000. (C) Virus production of eIF5A1-ORF carrying the eGFP can be visualized as green glow under a fluorescent microscope. (D) Vector information of shRNA target sequences for RSH047876-5-HIVmU6. (E) Virus production of eIF5A1-shRNA can be visualized as red glow under a fluorescent microscope.

Figure S2

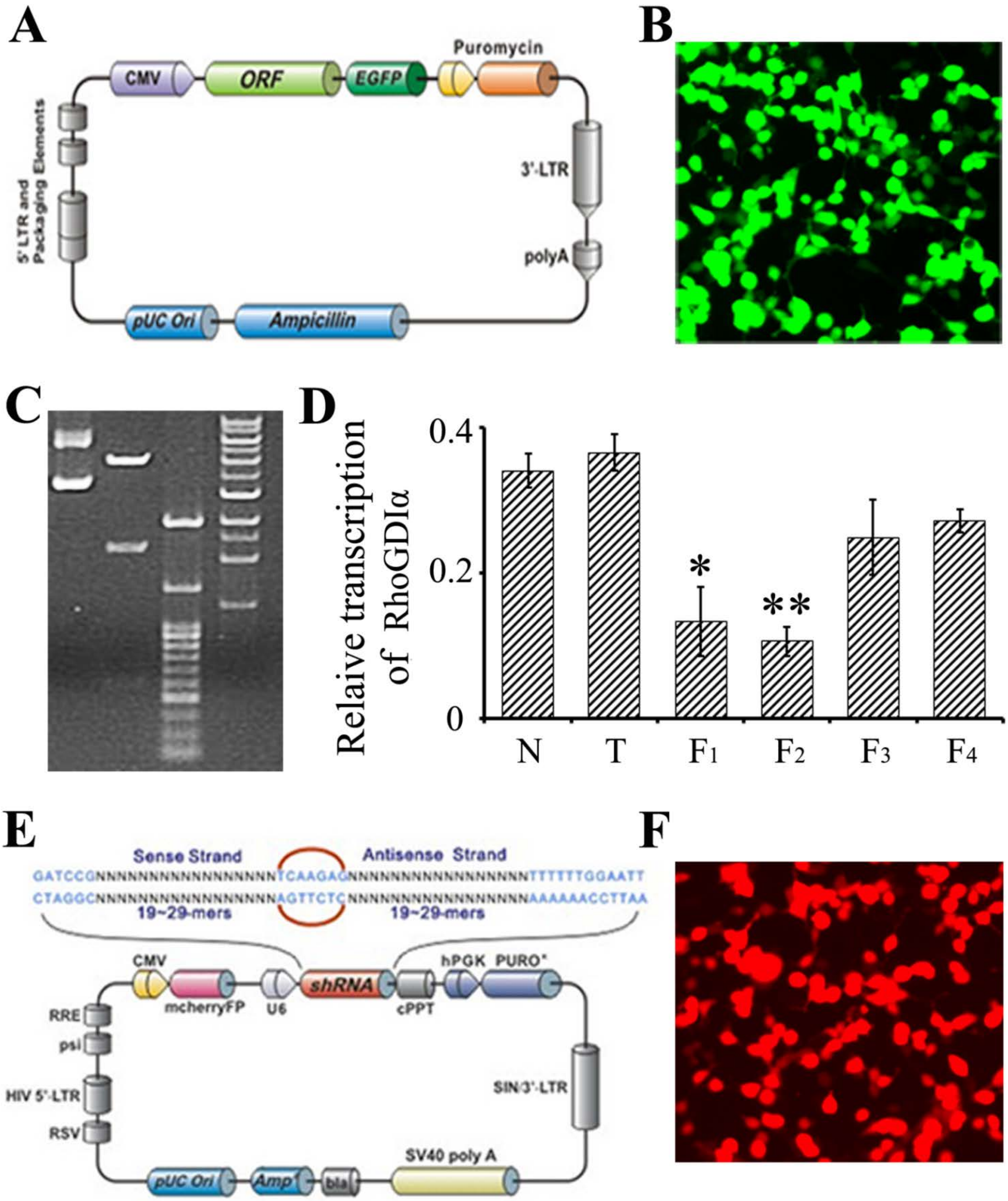

Figure S2. Packaging of recombinant RhoGDIα Lentivirus

(A) Vector information of ORF sequence for CCS-Rn10087-Lv122. (B) Virus production of RhoGDIα-ORF carrying the eGFP can be visualized as green glow under a fluorescent microscope. (C) Verify the correctness of Clone CCS-Rn10087-Lv122. CCS-Rn10087-Lv122 was digested by Acc65I, AsuII and NotI restriction enzyme and run on 1.0% agarose gel. Lane1:

CCS-Rn10087-Lv122 plasmid. Lane2: CCS-Rn10087-Lv122 plasmid digested. There are three expected bands (~5970/480/2181bp). Lane3: DNA Ladder 3000. Lane4: DNA Ladder 15000. (D) The effectiveness of shRNA fragments was compared by PCR in the PC12 cell line. N, normal cells; T, add transfection reagent into culture medium; F1-4, treatment with No.1-4 shRNA fragments, respectively; Results are reported as means ± SEM. $^{**}P<0.01$; $^{*}P<0.05$. (E) Vector information of shRNA target sequences for RSH045250-HIVmU6. (F) Virus production of RhoGDIα-shRNA can be visualized as red glow under a fluorescent microscope.

Supplemental tables

Table S1. Differential proteins identified by spectrum analysis.

No.	Protein name	Accession GI	PI/MW calculated	PI/MW observed	Score	Coverage	Fold-Change
1	Albumin	55391508	6.17/69200	6.09/68714	330	18%	2.60
2	Calreticulin	11693172	4.73/45800	4.33/47966	141	20%	4.90
3	Ubiquitin carboxy- terminal hydrolase L1	61098212	5.83/28900	5.14/24822	195	33%	2.75
4	Tumor rejection antigen gp96	58855966	5.13/77400	5.02/74162	253	18%	4.49
5	Rho GDP-dissociation inhibitor alpha	31982030	5.38/21820	5.12/23393	273	50%	1.86
6	Enolase 2, Gamma	26023949	5.10/48300	5.03/47111	396	27%	2.23
7	Aldolase C	6978489	6.39/47200	6.67/39259	238	25%	1.64
8	Glycerol-3-phosphate dehydrogenase1	57527919	5.98/38600	6.16/37428	129	17%	2.15
9	Triosephosphate isomerase-1	38512111	7.39/29630	7.07/26701	177	25%	5.64
10	Glutathione S- Transferase	25453412	6.32/29800	6.89/23424	172	38%	2.83
11	Eukaryotic translation initiation factor5A	4503545	4.76/22300	5.08/16821	160	31%	3.43
12	Cytochrome c oxidase, subunit Va	55992	6.23/13800	6.46/12681	64	34%	4.13
13	Fatty acid-binding Protein	1836058	5.98/14200	6.14/15035	153	22%	6.72
14	Alpha-enolase	56757324	5.77/48200	6.16/47098	288	18%	2.20
15	Annexin 1	6978501	6.47/40500	6.97/38805	269	37%	0.35
16	Dynein-like protein 9A	871917	3.84/46300		25		0.14
17	Glyceraldehyde-3- phosphate dehydrogenase	8393418	7.92/32100	8.14/35805	117	30%	0.30
18	Mitochondrial ribosomal protein L13	55741549	6.31/17600		28		0.12
19	Transferrin	149018747	6.46/71500	6.41/65110	66	12%	0.41
20	Vimentin	14389299	5.07/63500	5.06/53700	214	26%	0.37

Table S2. The PCR primers used in this study were listed in following table.

Gene	Forward primer	Reverse primer
Albumin	GCTGCTGACTTTGTTGAGG	CATATTTCTTAGCAAGTCTCAG
Calreticulin	CATCTGCGGTCCTGGCACCAAGAA	CCGACTCCACCTGGCTGTTGTCAA
Ubiquitin carboxy-terminal hydrolase L1	TACGAGCTCGATGGGCGAAT	TGAATTCTCTGCAGACCTT
Tumor rejection antigen gp96	TCTGCTGCGTCCTGCTGACCTT	TCATCGTCTGTCCGTGAGCCTTCT
Rho GDP-dissociation inhibitor alpha	GGAGTACCGGATAAAAATCT	ATGTAGTCAGTCTTGTCAA
Enolase 2, Gamma	CCACATCAACAGCACCATCGCACC	TGAGGTCGGAGTTCCCAGCCAGTT
Aldolase C	TTGTCCGCACCATCCAGGAGAAGG	GCACAGCGTTCCAAGAGTCCATCC
Glycerol-3-phosphate dehydrogenase 1	TCCGCATCACCGTGGTACAAGAGG	ATCACTGCCGCCTTGGTGTTGTCA
Triosephosphate isomerase -1	ACTCGGAGTGATCGCCTGCATTGG	GCAAGTCGCTCCAGTCACAGAACC
Glutathione S-Transferase	GAGACAGAGGAGGAGCGGATTCGT	TTTGGGAAGGCGTCCAGGCACTT
Eukaryotic translation initiation factor 5A	CAGCATTACGTAAGAATGGT	GACGGGCAGATATCTTCAT
Cytochrome c oxidase, subunit Va	CGGTTAAATGATTTTGCTAGT	CACTTTGTCAAGGCCCAGTT
Fatty acid-binding Protein	ACTGAGACGGTCTGCACCTTCACA	TGACGCACTCCACCACCATCTTCC
Alpha-enolase	GGCGGTTCTCATGCTGGCAACAAG	GCGAATCCACCCTCATCACCCACA
Annexin 1	ATGAACTCCGTGCTGCCATGAAGG	TGGCGAGAGCAAGCAAGGCATTAC
Dynein-like protein 9A	CAGGCACAGGCAAGACAGAGACCA	GCACAGCCACCACTGACAAGACCT
Glyceraldehyde-3-phosphate dehydrogenase	CCTCAAGATTGTCAGCAAT	CCATCCACAGTCTTCTGAGT
Mitochondrial ribosomal protein L13	CGGGTGGCTTCAGACAGGTAACA	GCATCATCGTTCTTCTGTGCAGGT
Transferrin	TGAGTGGAGCGTCAGCAGCAATGG	GCCTTCACCACAGCCACGGCATAA
Vimentin	TCAGACAGGATGTTGACAAT	GACATGCTGTTCCTGAATCT
β-actin	GAAGATCAAGATCATTGCTCCT	TACTCCTGCTTGCTGATCCA

Table S3 Animal grouping for proteomic analysis and BBB scores record

Group	Treatment	Laboratory procedures	BBB[#] scores
Sham	Sham	Cord taken from 8 rats for molecular analysis and other 8 rats for immunofluorescence analysis	8 rats tested at 0~28dpo
14dpo[#]	SCT[#] 14dpo	At 14dpo, caudal cord taken from 8 rats for proteomic and molecular analysis	
28dpo	SCT 28dpo	At 28dpo, caudal cord taken from 8 rats for proteomic and molecular analysis	8 rats tested at 0~28dpo

[#] BBB, Basso, Beattie, and Bresnahan; SCT, spinal cord transection at T8; dpo, days post-operation.

TableS4 Animal grouping to investigate the role of eIF5A1 in the function recovery and neuroplasticity

Group	Treatment	Laboratory procedures	BBB scores
S[#]	Sham	Cord taken from 8 rats for molecular analysis and other 8 rats for immunofluorescence analysis	16 rats tested at 0~28dpo
C[#]	Caudal cord of SCT rats injected with lentiviral empty vector	Caudal cord taken from 8 rats at 28dpo for molecular analysis and other 8 rats for immunofluorescence analysis	16 rats tested at 0~28dpo
F+[#]	Caudal cord of SCT rats injected with lentiviral of eIF5A1 overexpression	Caudal cord taken from 8 rats at 28dpo for molecular analysis and other 8 rats for immunofluorescence analysis	16 rats tested at 0~28dpo
F-[#]	Caudal cord of SCT rats injected with lentiviral of eIF5A1 interference	Caudal cord taken from 8 rats at 28dpo for molecular analysis and other 8 rats for immunofluorescence analysis	16 rats tested at 0~28dpo

[#] S: sham group; C: control group in which transfected lentiviral empty vector; F+: group of eIF5A1 overexpression in which transfected lentiviral plasmids encoding overexpressed sequence of eIF5A1; F-: group of eIF5A1 interference in which transfected lentiviral plasmids encoding interfering RNA sequence of eIF5A1.

TableS5 Animal grouping to determine the relationship between eIF5A1 and Rho GDIα in the functional recovery of SCT rats

Group	Treatment	Laboratory procedures	BBB scores
S[#]	Sham	Cord taken from 8 rats for molecular analysis and other 8 rats for immunofluorescence analysis	16 rats tested at 0~28dpo
C[#]	Lentiviral control vector injected into caudal cord of SCT rats	Caudal cord taken from 8 rats at 28dpo for molecular analysis and other 8 rats for immunofluorescence analysis	16 rats tested at 0~28dpo
F+G-[#]	Lentivirus of eIF5A1 overexpressed and Rho GDIα interfered, injected into caudal cord of SCT rats	Caudal cord taken from 8 rats at 28dpo for molecular analysis and other 8 rats for immunofluorescence analysis	16 rats tested at 0~28dpo
F-G+[#]	Lentivirus of eIF5A1 interfered and Rho GDIα overexpressed, injected into caudal cord of SCT rats	Caudal cord taken from 8 rats at 28dpo for molecular analysis and other 8 rats for immunofluorescence analysis	16 rats tested at 0~28dpo

[#] S: sham group; C: control group in which transfected lentiviral vector; F+G-: group of eIF5A1 overexpression combined Rho GDIα interference in which co-transfected lentiviral plasmids encoding overexpressed sequence of eIF5A1 and interfered fragment of Rho GDIα; F-G+: group of under-expressing eIF5A1 combined over-expressing Rho GDIα in which co-transfected lentiviral plasmids encoding interfered fragment of eIF5A1 and overexpressed sequence of Rho GDIα.

Supplemental mass spectrum data

Mass Spectrometry data S1

Mass Spectrometry information of eIF5A1 (NO.11 spot)

Table 1 list the potential protein that identified by Mass Spectrometry on 4700 Proteomics Analyzer. Eukaryotic translation initiation factor 5A (eIF5A1), which the Probability Based Mowse Score was 160 (greater than 56 means significant), was accepted; Table 2 shows 4 peptides of eIF5A1 were identified by Mass Spectrometry. The peptide mass fingerprinting (PMF) is as follows. Matched and no matched peaks are listed in Table 3. Tandem mass spectrometry (MS/MS) confirmed the "2580.39" peptide (87-109), the ions score is 135. For each PMF and MS/MS picture, we could use mouse to drag the pictures to their clearly size.

Table 1. List of potential protein (No.1 was accepted)

	Accession	Mass	Score	Description
1.	gi|76096304	16821	160	eukaryotic translation initiation factor 5A1
2.	gi|19424164	21542	27	interleukin 11
3.	gi|206705	10738	24	ornithine aminotransferase
4.	gi|19570809	33460	23	gamma-adaptin
5.	gi|60422784	54081	23	Dncli2 protein
6.	gi|62645226	37062	22	PREDICTED: similar to methionine aminopeptidase-like 1
7.	gi|400922	28332	22	Transcription initiation factor IIF subunit beta
8.	gi|31542922	28374	22	general transcription factor IIF, polypeptide 2
9.	gi|6942219	19272	22	thioredoxin reductase 1
10.	gi|13591938	54711	22	dynein, cytoplasmic, light intermediate polypeptide 2

Table 2. The details about the eIF5A1 are showed as follows.

Observed	Mr(expt)	Mr(calc)	Delta	Start	End	Miss	Ions score	Peptide
1298.80	1297.79	1297.74	0.05	56	67	0	---	K.VHLVGIDIFTGK.K
1426.91	1425.91	1425.83	0.07	56	68	1	---	K.VHLVGIDIFTGKK.Y
1435.82	1434.82	1434.73	0.08	35	47	1	---	K.GRPCKIVEMSTSK.T
2580.39	2579.38	2579.29	0.09	87	109	0	135	R.NDFQLIGIQDGYLSLLQDSGEVR.E

Table 3. The matched information about the eIF5A1 from PMF

No matched	Matched peptides shown in Bold Red
700.39,712.31,754.39,802.35,803.67,804.33,804.33,818.35,832.37,834.37,83	**1** MADDLDFETG DAGASATFPM
4.37,846.35,848.36,849.37,854.34,870.32,879.58,886.30,886.30,923.61,967.	**21** QCSALRKNGF VVLK**GRPCKI**
62,1011.68,1249.80,1293.83,1337.85,1353.85,1365.90,1381.90,1397.87,1406	**31** **VEMSTSK**TGK HGHAK**VHLVG**
.75,1409.90,1425.92,1439.87,1441.88,1453.96,1469.95,1485.91,1513.97,151	**41** **IDIFTGKK**YE DICPSTHNMD
3.97,1529.95,1541.99,1557.99,1570.03,1573.97,1586.03,1602.03,1602.03,16	**51** VPNIKR**NDFQ LIGIQDGYLS**
18.00,1630.09,1634.00,1642.07,1646.05,1662.02,1674.10,1690.09,1706.06,1	**61** **LLQDSGEVR**E DLRLPEGDLG

722.05,1734.12,1750.09,1778.14,1794.13,1806.16,1828.26,1922.03,2626.42	**71** KEIEQKYDCG EEILITVLSA
	**81** MTEEAAVAIK AMAK

PMF

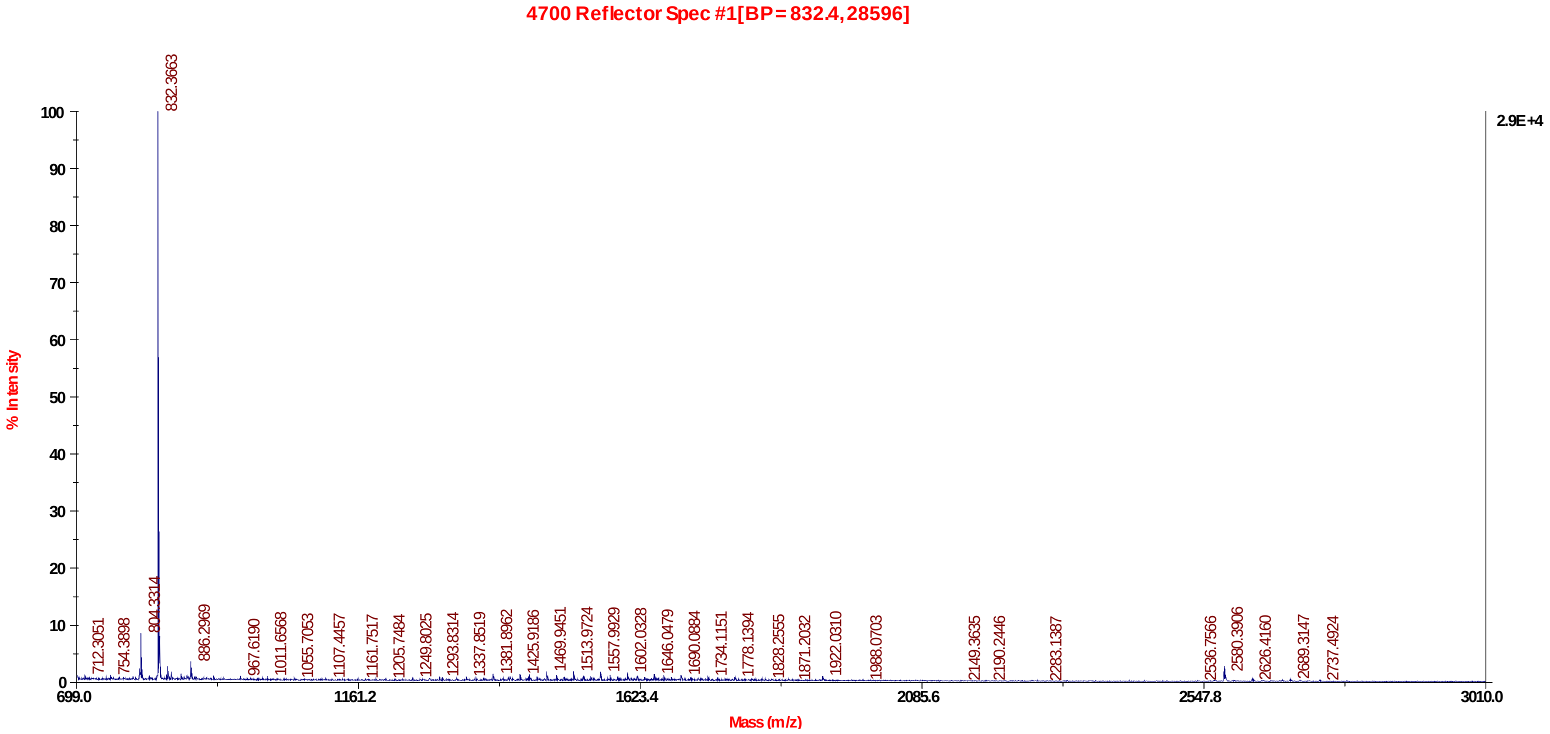

MS/MS

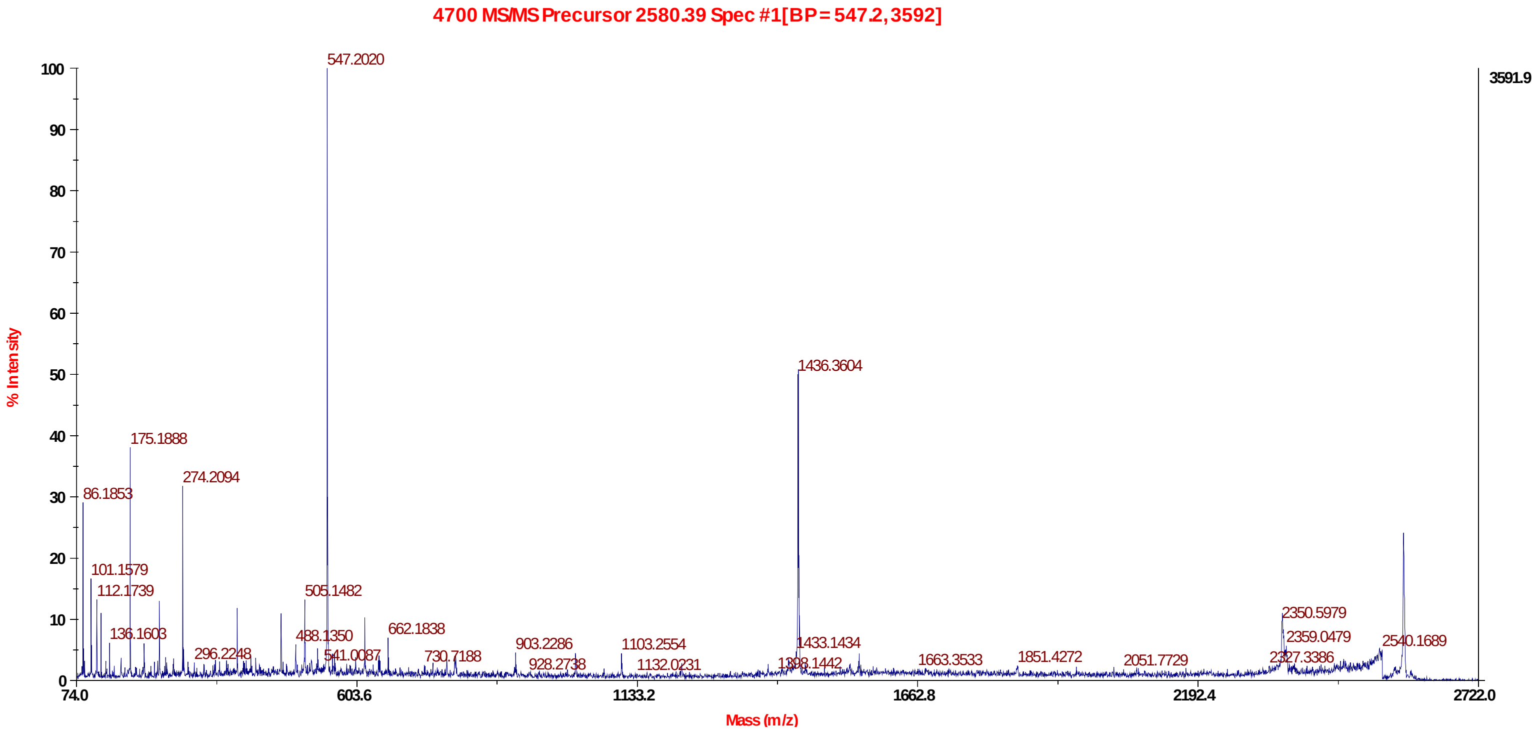

4700 MS/MS Precursor 2580.39 Spec #1[BP = 547.2, 3592]
% Intensity
Mass (m/z)
100
90
80
70
60
50
40
30
20
10
0
74.0
603.6
1133.2
1662.8
2192.4
2722.0
3591.9
547.2020
1436.3604
175.1888
274.2094
86.1853
101.1579
112.1739
505.1482
136.1603
488.1350
662.1838
296.2248
541.0087
730.7188
903.2286
928.2738
1103.2554
1132.0231
1433.1434
1398.1442
1663.3533
1851.4272
2051.7729
2350.5979
2359.0479
2327.3386
2540.1689

Mass Spectrometry data S2

Mass Spectrometry information of RhoGDIα (NO.5 spot)

Table 1 list the potential protein that identified by Mass Spectrometry on 4700 Proteomics Analyzer. Rho GDP dissociation inhibitor alpha (RhoGDIα) , which the Probability Based Mowse Score was 237 (greater than 56 means significant), was accepted. Table 2 shows 11 peptides of RhoGDIα were identified by Mass Spectrometry. The peptide mass fingerprinting (PMF) is as follows. Matched and no matched peaks are listed in Table 3. Tandem mass spectrometry (MS/MS) confirmed three peptides “980.53” (128-134), “1601.79” (139-152), “1650.96” (59-74), the ions’ scores was 26, 60, and 35 respectively. For each PMF and MS/MS picture, we could use mouse to drag the pictures to their clearly size.

Table 1. List of potential protein (No.1 was accepted).

	Accession	Mass	Score	Description
1.	gi|31982030	23393	237	Rho GDP dissociation inhibitor (GDI) alpha
2.	gi|55926133	38632	43	replication factor C 2 (40kD)
3.	gi|62664490	48212	42	PREDICTED: similar to immunity-related GTPase family, cinema 1
4.	gi|442368	52684	42	neuronal olfactomedin-related ER localized protein
5.	gi|59891429	48160	42	hypothetical protein LOC307414
6.	gi|17865343	55407	38	olfactomedin 1
7.	gi|2745840	185810	37	postsynaptic density protein; citron
8.	gi|6679593	24954	35	RAB3A, member RAS oncogene family
9.	gi|62078867	30271	34	hypothetical protein LOC500528
10.	gi|12018232	14880	34	lectin, galactose binding, soluble 7

Table 2. The details about the RhoGDIα are showed as follows.

Observed	Mr(expt)	Mr(calc)	Delta	Start	End	Miss	Ions score	Peptide
734.38	733.38	733.32	0.05	44	49	0	---	K.DDESLR.K
752.39	751.38	751.35	0.03	106	111	0	---	K.EGVEYR.I
949.58	948.57	948.54	0.03	51	58	1	---	K.YKEALLGR.V
980.53	979.52	979.49	0.03	128	134	0	26	K.YIQHTYR.K
1108.69	1107.68	1107.58	0.10	128	135	1	---	K.YIQHTYRK.G
1245.59	1244.59	1244.55	0.04	142	152	0	---	K.TDYMVGSYGPR.A
1601.79	1600.79	1600.76	0.03	139	152	1	60	K.IDKTDYMVGSYGPR.A
1650.96	1649.95	1649.91	0.04	59	74	0	35	R.VAVSADPNVPNVIVTR.L
1783.87	1782.86	1782.80	0.06	153	167	0	---	R.AEEYEFLTPMEEAPK.G
1917.98	1916.98	1916.93	0.04	34	49	1	---	K.SIQEIQELDKDDESLR.K
2364.12	2363.12	2363.11	0.01	181	199	1	---	R.FTDDDKTDHLSWEWNLTIK.K

Table 3. The matched information about the RhoGDIα from PMF

No matched	Matched peptides shown in **Bold Red**
712.28,770.45,804.30,806.31,831.33,83	**1** MAEQEPTAEQ LAQIAAENEE DEHSVNYKPP AQK**SIQEIQE**
2.34,832.34,848.34,854.32,870.30,876.	**41** **LDKDDESLR**K **YKEALLGRVA VSADPNVPNV IVTR**LTLVCS
30,881.29,988.54,1002.51,1120.68,113	**81** TAPGPLELDL TGDLESFKKQ SFVLK**EGVEY R**IKISFRVNR
2.73,1316.70,1556.76,1623.81,1672.94,	**121** EIVSGMK**YIQ HTYRK**GVK**ID KTDYMVGSYG PRAEEYEFLT**
1738.81,1797.89,1931.99,2008.97,2046	**161** **PMEEAPK**GML ARGSYNIKSR **FTDDDKTDHL SWEWNLTIK**K
.08,2060.10,2492.30,2494.21,2704.51	**201** EWKD

PMF

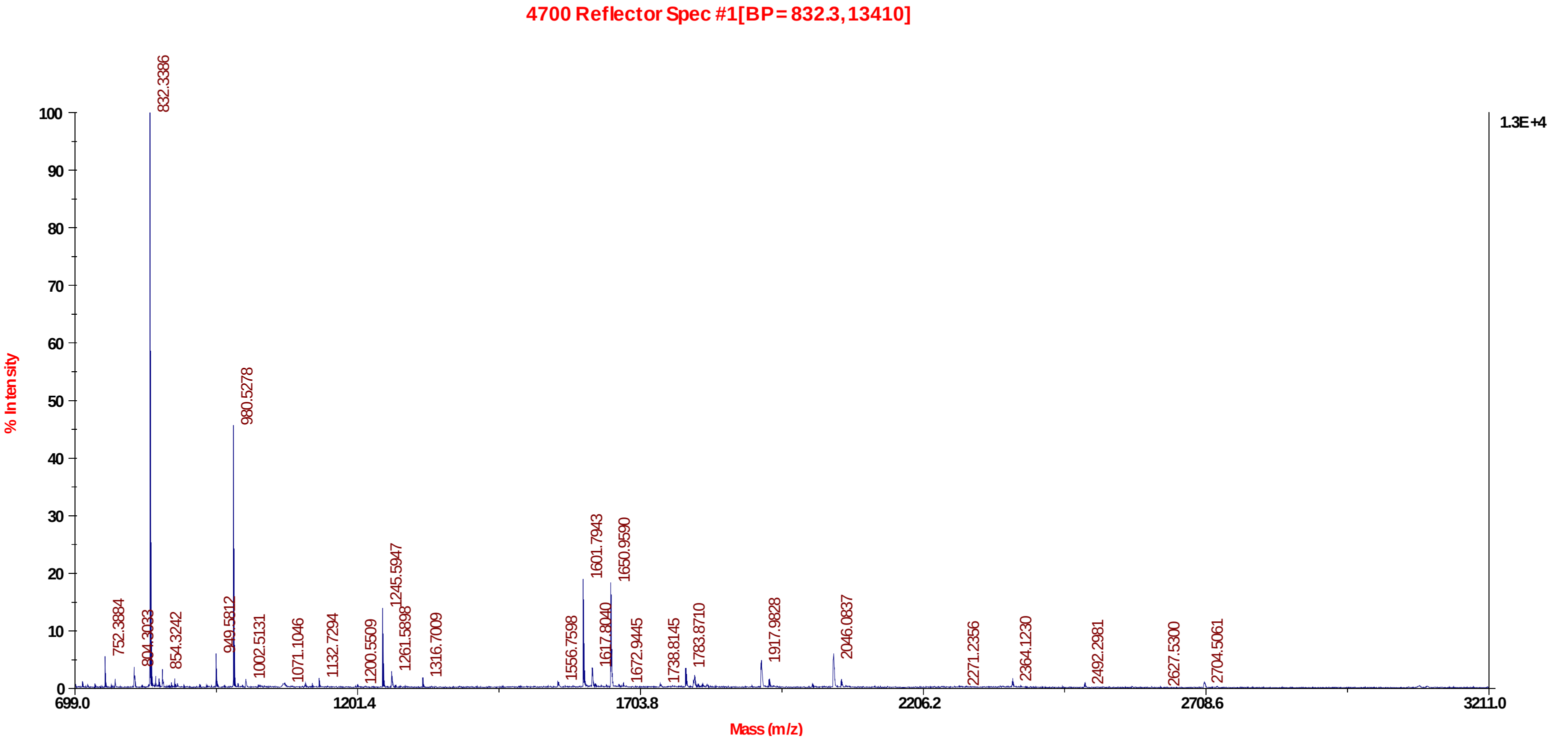

MS/MS

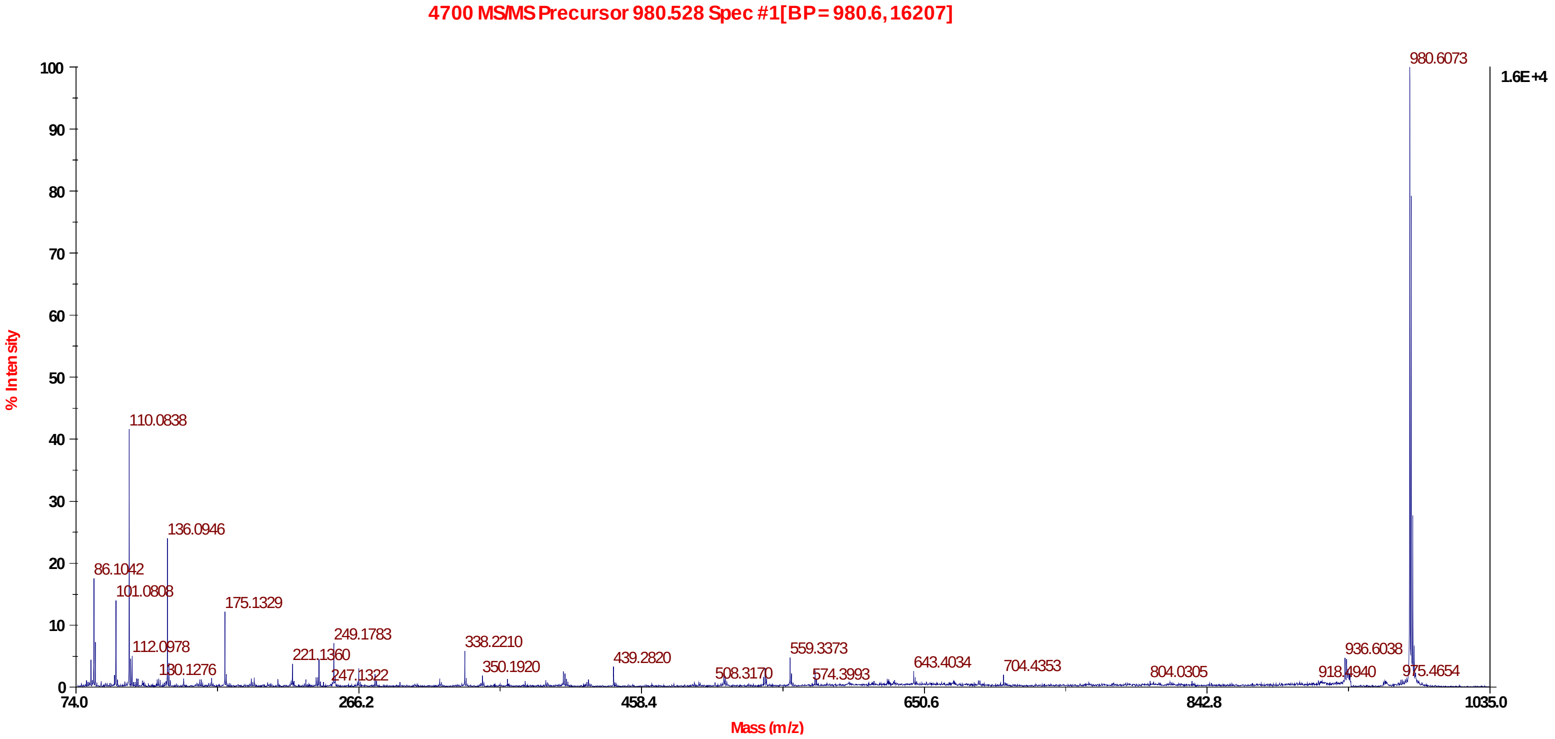
4700 MS/MS Precursor 980.528 Spec #1[BP = 980.6, 16207]
% Intensity
Mass (m/z)
1.6E+4
980.6073
110.0838
136.0946
86.1042
101.0808
175.1329
112.0978
130.1276
249.1783
221.1360
247.1322
338.2210
350.1920
439.2820
508.3170
559.3373
574.3993
643.4034
704.4353
804.0305
936.6038
918.4940
975.4654
74.0
266.2
458.4
650.6
842.8
1035.0

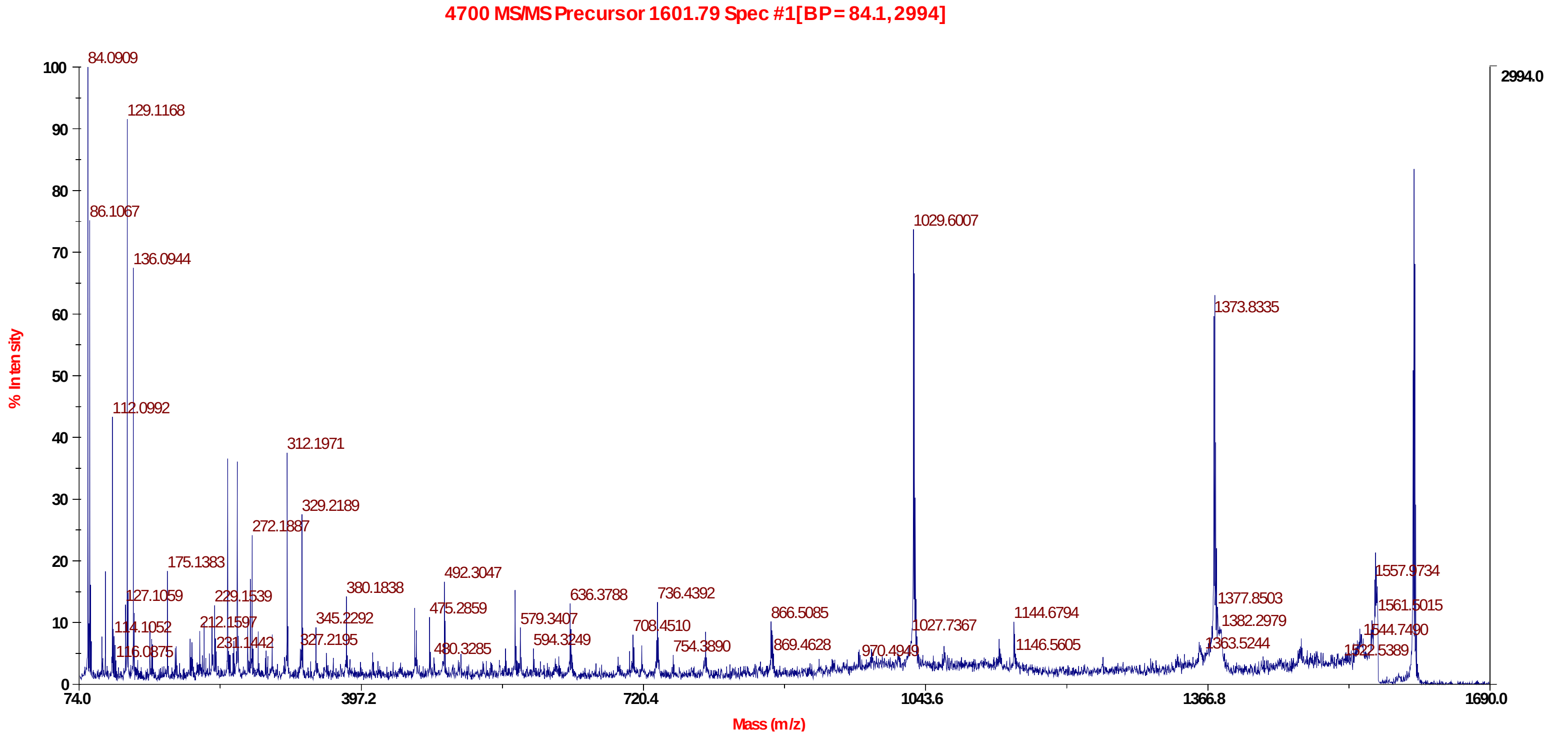
4700 MS/MS Precursor 1601.79 Spec #1[BP = 84.1, 2994]
% Intensity
Mass (m/z)
2994.0
84.0909
129.1168
86.1067
136.0944
112.0992
312.1971
329.2189
272.1887
175.1383
127.1059
229.1539
114.1052
212.1597
116.0875
231.1442
380.1838
345.2292
327.2195
492.3047
475.2859
480.3285
579.3407
594.3249
636.3788
708.4510
736.4392
754.3890
866.5085
869.4628
970.4949
1029.6007
1027.7367
1144.6794
1146.5605
1373.8335
1377.8503
1382.2979
1363.5244
1557.9734
1561.5015
1544.7490
1522.5389
74.0
397.2
720.4
1043.6
1366.8
1690.0

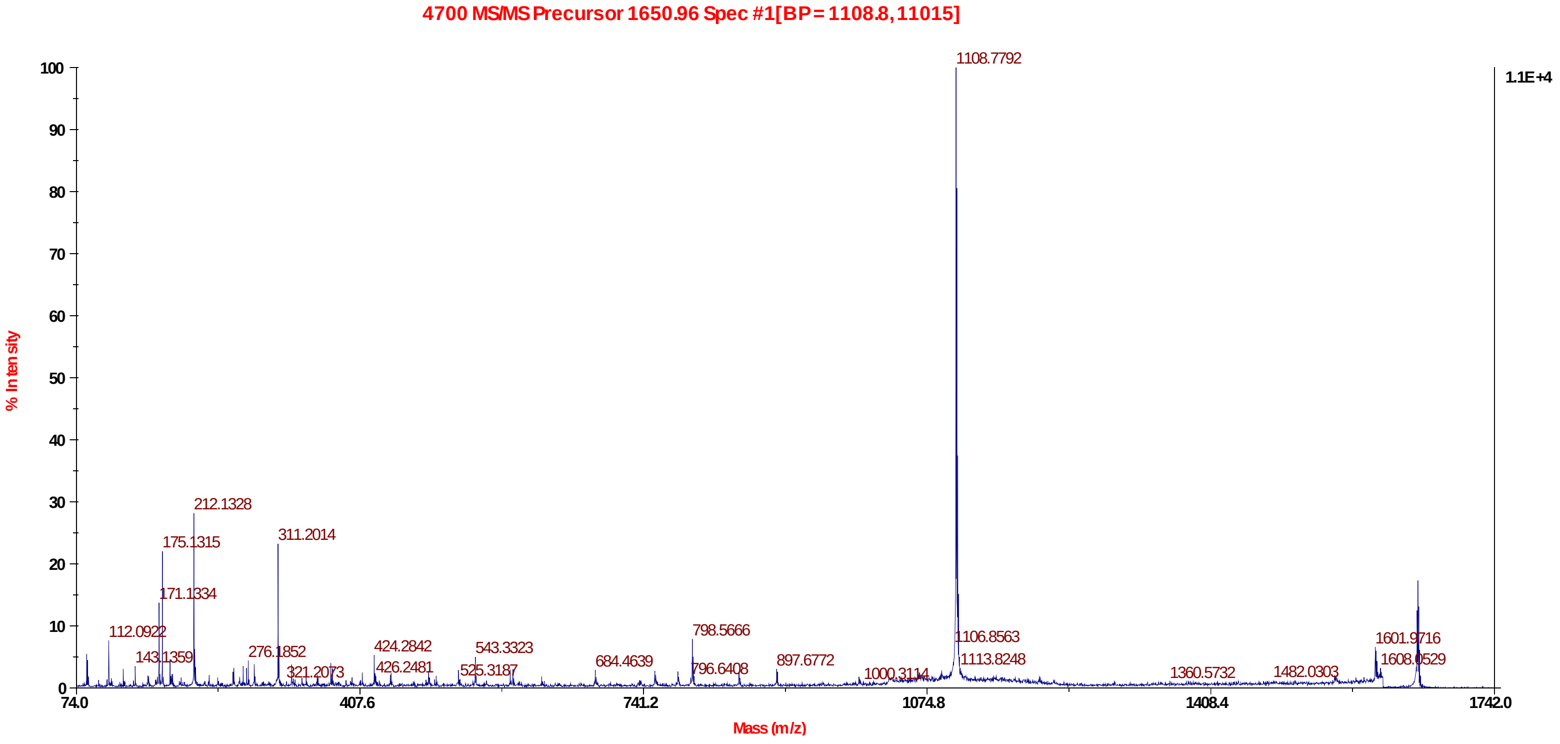
4700 MS/MS Precursor 1650.96 Spec #1[BP = 1108.8, 11015]
% Intensity
Mass (m/z)
1.1E+4
1108.7792
212.1328
175.1315
311.2014
171.1334
112.0922
143.1359
276.1852
321.2073
424.2842
426.2481
543.3323
525.3187
684.4639
798.5666
796.6408
897.6772
1000.3114
1106.8563
1113.8248
1360.5732
1482.0303
1601.9716
1608.0529
74.0
407.6
741.2
1074.8
1408.4
1742.0
